# Thermal Control of Material Placement in Heterostructured Metal Sulfide Nanorods

**DOI:** 10.1021/acs.chemmater.6c00154

**Published:** 2026-05-06

**Authors:** Chul-Hyun Jeong, Raymond E. Schaak

**Affiliations:** † Department of Chemistry, 8082 The Pennsylvania State University, University Park, Pennsylvania 16802, United States; ‡ Department of Chemical Engineering, The Pennsylvania State University, University Park, Pennsylvania 16802, United States; § Materials Research Institute, The Pennsylvania State University, University Park, Pennsylvania 16802, Unites States

## Abstract

A hallmark of nanoparticle cation exchange reactions is their ability to deterministically place different materials at precise locations within the same nanoparticle. This synthetic control underpins a broad application space, with synergistic properties that emerge from the relative spatial arrangements of the materials. However, despite advances in our understanding of how and why certain arrangements of materials and interfaces are preferentially observed as products of cation exchange reactions, our knowledge of design guidelines and synthetic tuning knobs remains limited. Here, we correlate the placement of ZnS, CdS, Co_9_S_8_, and Cu_1.8_S segments within heterostructured nanorods with the superionic transition temperature of copper sulfide, which is the most common nanoparticle template used for cation exchange. For cation exchange reactions that occur below the superionic transition temperature of approximately 100 °C, the resulting materials appear at various locations with the nanorods, including at the tips and within the body region. However, at higher cation exchange temperatures, material placement at the nanorod tips is observed almost exclusively. Additionally, we observe thin regions of copper sulfide sandwiched between Co_9_S_8_ and both ZnS and CdS, which help to relieve interfacial lattice strain. Overall, many different heterostructured nanorod architectures can be accessed through simple temperature-controlled partial cation exchange reactions that leverage interrelated contributions from interfacial lattice strain and the superionic nature of copper sulfide.

## Graphical abstract

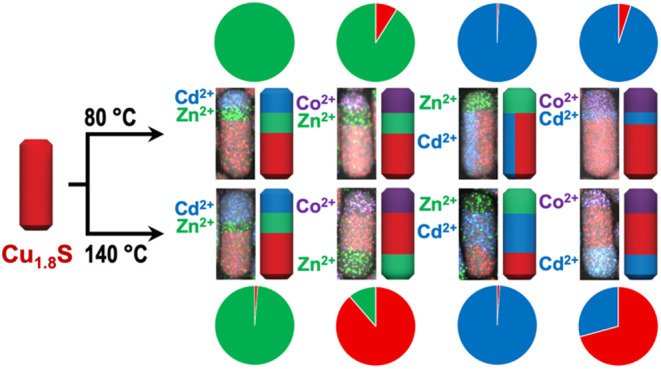

## Introduction

Heterostructured nanoparticles, which contain two or more distinct materials, can additively or synergistically combine the properties of each material to function in ways that are unachievable in single-composition nanoparticles.^1–8^ This functional versatility and high compositional tunability makes heterostructured nanoparticles promising materials for applications that include photocatalysis,^9^ optoelectronics,^10^ thermoelectrics,^11^ and energy conversion^12,13^ and storage.^14^ Cation exchange reactions can be used to synthesize complex heterostructured nanoparticles by replacing some of the cations in a template nanoparticle with incoming cations from solution.^15,16^ For example, nanoparticles of roxbyite copper sulfide (Cu_1.8_S) can transform to products such as ZnS, CdS, and Co_9_S_8_ upon exchanging the Cu^+^ cations with Zn^2+^, Cd^2+^, and Co^2+^, respectively.^17–20^ Partial cation exchange reactions, which only replace a fraction of the Cu^+^ cations in Cu_1.8_S, can produce heterostructured nanoparticles such as ZnS–Cu_1.8_S, CdS–Cu_1.8_S, and Co_9_S_8_–Cu_1.8_S, among others.^1,20–24^

Nanoparticle cation exchange reactions can be driven and controlled by considering the roles of hard–soft acid–base (HSAB) interactions, solvation effects, and other energetic contributors, as well as the presence of diffusion pathways within the nanoparticles.^15,16,24–27^ These considerations, along with crystallographic relationships, enable the sequential application of multiple partial cation exchange reactions to deterministically place different materials at precise locations within the same nanoparticle, leading to tunable heterostructuring.^24,28^ Heterostructuring occurs because once cations enter a host nanoparticle, they form separate phases that are immiscible with the host, and the immiscible phases are necessarily separated by interfaces.^1,24,29^ The interfaces that form are those that minimize strain, and therefore that exhibit the best lattice matching.^22,24,30,31^ Despite this knowledge, rigorous control over the spatial arrangements of the constituent materials in heterostructured nanoparticles, as well as the interfaces that connect them, can be challenging to achieve in practice.

Current design guidelines predict that each successive partial cation exchange reaction in a multiexchange sequence will install a material adjacent to the material that was most recently exchanged.^24^ For example, if a partial Co^2+^ exchange reaction is applied to a ZnS–Cu_1.8_S nanorod, existing knowledge predicts that the product of a subsequent M^2+^ exchange reaction will locate between the ZnS and Cu_1.8_S domains, forming ZnS–M*_x_* S–Cu_1.8_S. However, products such as ZnS–Cu_1.8_S–M_*x*_S are sometimes observed instead,^32^ indicating that our existing design guidelines have limitations. It is therefore important to better understand how and why interfaces form, and where different materials locate, during partial cation exchange reactions that produce heterostructured nanoparticles.

Temperature is emerging as an important variable that influences the formation of the intraparticle frameworks that define heterostructuring during partial cation exchange reactions.^33,34^ Vacancy-rich copper sulfide phases, including Cu_1.8_S that is used as a template for nanoparticle cation exchange, exhibit a superionic transition around 100 °C.^34–37^ Below the superionic transition temperature, the Cu^+^ cations reside in their expected crystallographic sites, but above this temperature, the Cu^+^ cations can easily migrate among all possible sites. This “cationic liquid” state facilitates rapid diffusion, and regions of other sulfide phases that are embedded within a Cu_1.8_S nanoparticle can quickly migrate to find their energetically preferred configurations. For example, partial Zn^2+^ exchange of Cu_1.8_S nanorods under certain conditions can produce small patches of ZnS within the nanorods. Upon heating these patchy ZnS–Cu_1.8_S nanorods above the superionic transition temperature, the ZnS patches can migrate, relocate, and coalesce to minimize interfacial energy. During this thermally induced process, the ZnS patches often converge at nanorod tips, which minimizes both the interfacial energies and the number of interfaces between the immiscible ZnS and Cu_1.8_S phases.^34^

Lattice strain and interfacial energy clearly play key roles in rationalizing the formation and location of material domains in heterostructured nanoparticles formed through cation exchange, and as a result have received the most attention.^22,24,30,31,38^ However, the influence of reaction temperature on interface formation and material placement within cation-exchanged heterostructured nanoparticles remains largely unknown, despite the role of the superionic transition in the commonly used Cu_1.8_S nanoparticle template.^34^ Here, we show that the temperature of the cation exchange reaction relative to the temperature of the superionic transition in Cu_1.8_S defines how the materials within heterostructured metal sulfide nanoparticles migrate and configure, both individually and coupled with other materials. Superionic transitions, lattice considerations, and partial cation exchange reactions ultimately allow nuanced control over heterostructuring in multicomponent metal sulfide nanorods, which provide an expanded set of design guidelines for controlling precise material placement within heterostructured nanomaterials.

## Experimental Section

For all syntheses and preparations of cation solutions, a standard Schlenk line setup was employed using a 50 mL three-neck round-bottom flask equipped with a heating mantle, gas flow adapter, reflux condenser, thermometer adapter, glass-encapsulated thermocouple, rubber septum, and magnetic stir bar. Standard Schlenk line techniques were used to ensure an air-free environment and prevent copper sulfide etching reactions.^33,39,40^ Temperature control was achieved using a J-KEM Scientific temperature controller, unless otherwise stated.

### Chemicals

Copper­(II) nitrate trihydrate [Cu­(NO_3_)_2_·3H_2_O, puriss. p.a. 99–104%], trioctylphosphine oxide [TOPO, 99%], 1-octadecene [ODE, technical grade, 90%], oleylamine [OLAM, technical grade, 70%], tert-dodecanethiol [t-DDT, mixture of isomers, 98.5%], 1-dodecanethiol [1-DDT, ≥98%], zinc­(II) chloride [ZnCl_2_, anhydrous, ACS reagent grade, ≥97%], and cobalt­(II) chloride [CoCl_2_, 97%] were purchased from Sigma-Aldrich. Cadmium­(II) chloride [CdCl_2_, anhydrous, trace metal basis, 99.99%] was purchased from Alfa Aesar. Benzyl ether [BE, 99%] was purchased from Acros Organics. Trioctylphosphine [TOP, >85%] was purchased from TCI America. All solvents (hexanes, toluene, acetone, and isopropyl alcohol [IPA]) were of analytical grade. All the above chemicals were used as received without further purification.

### Preparation of 0.015 M Cation Stock Solutions

All cation solutions were prepared following a modification of published procedures.^33^ The weighed amounts of each metal chloride salt and the corresponding solvent volumes are listed in Table S1. The specific quantities of solvent and salt were combined in a 50 mL three-neck round-bottom flask under the standard Schlenk line setup. The flask was placed under vacuum and heated to 100 °C with stirring, maintaining this temperature for 30 min to degas the solution. After three cycles of vacuum and Ar, the flask was placed under a blanket of Ar. The temperature then increased to the value indicated in Table S1 and held there for 60 min, allowing the metal salt to fully dissolve in the organic solution. Next, the heating mantle was removed, and the flask was cooled to approximately 50 °C. Finally, the solution was collected in a 40 mL septum-capped vial for storage under ambient conditions, ready for cation exchange reactions.

### Partial Cation Exchange Reactions of Zn^2+^, Cd^2+^, and Co^2+^

For this section, we describe a generalized protocol for partial cation exchange, adapted from published procedures with minor modifications.^29^ The “cation solution” here refers generically to either a ZnCl_2_, CdCl_2_, or CoCl_2_ solution. In each reaction, we targeted an exchange fraction of 1/6 of the number of Cu^+^ cations in the Cu_1.8_S nanorods, using 0.015 M cation solution (0.96 mL). Temperatures between 80 and 140 °C were used for all cations; for the partial Cd^2+^ exchange, 60 °C was used as well. As a general procedure, benzyl ether (5 mL), OLAM (2 mL), ODE (1 mL), and 0.015 M cation solution (0.96 mL) were added to a 50 mL three-neck round-bottom flask under the standard Schlenk line setup. The flask was placed under vacuum with stirring, heated to 100 °C, and degassed for 30 min. Meanwhile, 14 mg of Cu_1.8_S nanorods were combined with TOP (3 mL) in a 20 mL septum-capped vial, cycled three times between vacuum and Ar, backfilled with Ar, then sonicated for 45 min to ensure complete dispersion. Once the flask had been degassed, it was cycled three times between vacuum and Ar, then kept under a blanket of Ar. The temperature was adjusted to one of the chosen set points (60 °C, 80 °C, 100 °C, 120 °C, or 140 °C) and held there to be ready for the exchange reaction. The sonicated Cu_1.8_S/TOP suspension was rapidly injected into the flask, and the reaction was allowed to proceed for 7 min at the selected temperature. Afterward, the flask was removed from the heating mantle and cooled in an ice bath to approximately 10 °C. The product was then transferred to centrifuge tubes containing a 1:1 (v/v) mixture of IPA:acetone, which had been precooled in an ice bath. After centrifugation at 14,500 rpm (18,335*g*) for 2 min, the supernatant was removed. The remaining precipitate was resuspended in toluene with brief sonication, subsequently washed with the IPA:acetone mixture, followed by one more centrifugation, and then this process was repeated once more, resulting in a total of three washes. During the final wash, a drop of OLAM was introduced just before resuspending the product in toluene to improve surface ligand coverage. After completing the three washes, the final product was suspended in hexanes and stored in a 20 mL vial under ambient conditions for further characterization. By varying the temperature (60 °C, 80 °C, 100 °C, 120 °C, or 140 °C) and incoming cation (Zn^2+^, Cd^2+^, or Co^2+^), we synthesized 13 samples (Table S2). Particle count numbers used for statistical analysis of heterostructure populations are based on a single reaction batch at each temperature for each cation.

### Sequential Cation Exchange Reactions

In this section, we describe a generalized sequential (two-step) cation exchange, using a modified published procedure.^29^ The “1^st^ cation” and “2^nd^ cation” solutions were selected from 0.015 M cation solutions (ZnCl_2_, CdCl_2_, or CoCl_2_), according to the intended injection sequence. For both sequences, 1/6 exchanges (relative to the number of Cu^+^ cations in Cu_1.8_S) were targeted, and a volume of 0.96 mL was used for each sequence. As a representative procedure, benzyl ether (5 mL), OLAM (2 mL), ODE (1 mL), and the 1^st^ cation solution (0.96 mL) were combined in a 50 mL three-neck round-bottom flask under the standard Schlenk line setup. The flask was placed under vacuum with stirring, heated to 100 °C, and degassed for 30 min. Meanwhile, TOP (3 mL) was combined with 14 mg of Cu_1.8_S nanorods in a 20 mL septum-capped vial. This vial was then cycled three times between vacuum and Ar, kept under Ar, and sonicated for 45 min to disperse the nanorods. Once the flask was fully degassed, it was cycled three times between vacuum and Ar, then maintained under a blanket of Ar. The temperature of the flask was adjusted to one of the chosen set points (80 °C or 140 °C) and held there in preparation for the sequential exchanges. The Cu_1.8_S/TOP suspension was rapidly injected into the flask, allowing the 1^st^ exchange to proceed for 7 min. Next, the 2^nd^ cation solution (0.96 mL) was injected under the same temperature, and the reaction continued for an additional 7 min. The flask was then placed in an ice bath to be cooled to approximately 10 °C. The product was transferred to centrifuge tubes containing a 1:1 (v/v) mixture of IPA:acetone, which had been cooled in an ice bath, followed by centrifugation at 14,500 rpm (18,335*g*) for 2 min. The supernatant was discarded, and the precipitate was re-dispersed in toluene with brief sonication, followed by addition of the IPA/acetone mixture. The suspension was then centrifuged, and these wash cycles were repeated for a total of three times. During the final wash, one drop of OLAM was added prior to re-dispersing in toluene, aiding ligand coverage. After completing all washes, the final product was suspended in hexane and stored in a 20 mL vial under ambient conditions for further characterization. The combinations of varying the temperature (80 °C or 140 °C), incoming cation (Zn^2+^, Cd^2+^, or Co^2+^), and injection sequence (1^st^ or 2^nd^) yielded 12 samples (Table S3). Particle count numbers used for statistical analysis of heterostructure populations are based on a single reaction batch at each temperature for each cation injection sequence.

### Characterization

Powder X-ray diffraction (XRD) data were collected using a Malvern Panalytical Empyrean diffractometer equipped with a Cu Kα radiation source. XRD data for cobalt-containing samples were acquired using a 1Der detector. Samples were prepared by drop-casting on zero-background silicon (Si) holders. CrystalMaker and CrystalDiffract (CrystalMaker Software Ltd., Oxford, UK) were used to simulate crystal structures and generate reference powder diffraction patterns. High-resolution transmission electron microscopy (HRTEM), high-angle annular dark-field scanning transmission electron microscopy (HAADF-STEM), and STEM energy-dispersive X-ray spectroscopy (STEM-EDS) data were acquired on a FEI Talos F200X S/TEM operated at 200 kV. STEM-EDS element maps were processed and analyzed using Thermo Scientific Velox software, and the EDS lines used for element mapping were Cu Kα, Zn Kα, Cd Lα, and Co Kα.

## Results and Discussion

To investigate how reaction temperature influences domain formation via cation exchange on roxbyite Cu_1.8_S nanorods (55 ± 4 nm × 20 ± 1 nm, Figure S1), we selected Zn^2+^, Cd^2+^, and Co^2+^ as the incoming cations. These cations were chosen to span a wide range of lattice strains and interfacial configurations. When Zn^2+^ and Cd^2+^ exchange with Cu^+^ in Cu_1.8_S nanorods, wz-ZnS and wz-CdS form (wz = wurtzite) because the distorted hexagonal close packed (hcp) sulfur sublattice of Cu_1.8_S is maintained during these cation exchange reactions (the sulfur sublattice of wurtzite is hcp).^19^ To minimize interfacial strain, partial cation exchange reactions with Zn^2+^ produce regions of wz-ZnS interfaced with Cu_1.8_S perpendicular to the long axis of the nanorods (Figure S2), while partial exchanges with Cd^2+^ produce wz-CdS interfaced with Cu_1.8_S parallel to the long axis of the nanorods (Figure S3).^28^ These configurations arise from crystal structure relationships, as discussed in the Supporting Information. In contrast, Co^2+^ exchange reactions on Cu_1.8_S nanorods typically produce Co_9_S_8_ instead of wz-CoS. Co_9_S_8_ has a cubic close packed (ccp) sulfur sublattice and therefore requires a series of lateral shifts to transform the sulfur sublattice from distorted hcp in Cu_1.8_S to ccp in Co_9_S_8_.^18,20^ We confirmed that partial Co^2+^ exchange on Cu_1.8_S nanorods produces Co_9_S_8_ at temperatures as low as 60 °C (Figure S4), which is well below the superionic transition temperature of Cu_1.8_S. We chose exchange temperatures of 80 °C, 100 °C, 120 °C, and 140 °C for the Zn^2+^, Cd^2+^, and Co^2+^ cations because these temperatures straddle the superionic transition temperature of Cu_1.8_S. For Cd^2+^, we additionally chose an exchange temperature of 60 °C because Cd^2+^ exchange reactions proceed more readily than Zn^2+^ and Co^2+^, enabling us to explore a wider temperature range compared to the other cations. For all experiments, we investigated the products by analyzing high-angle annular dark field (HAADF) images and scanning transmission electron microscopy-energy dispersive spectroscopy (STEM-EDS) element maps. Partial exchange reactions targeted an exchange fraction of 1/6 of the Cu^+^ cations in the Cu_1.8_S nanorods and quantitative STEM-EDS analysis confirmed the expected levels of exchange: 12.7% for Zn^2+^, 16.1–21% for Cd^2+^, and 10.9–11% for Co^2+^ (Table S4).

Analysis of the partial Zn^2+^ exchange reactions at each of the temperatures shows that the ZnS regions are interfaced with copper sulfide along the *a*-axis direction, which is perpendicular to the long axis of the nanorods (Table S2). This interface behavior is expected, because it corresponds to the crystallographic directions that minimize lattice mismatch between ZnS and Cu_1.8_S (Figure 1 and Table S5).^28^ However, statistical analysis of populations of individual nanorods at different temperatures reveal differences in the locations of the ZnS regions. Figure 2 shows four distinct types of ZnS/Cu_1.8_S heterostructured nanorods that are observed; these nanorods can be described as Cu_1.8_S with ZnS at one tip, ZnS at both tips, ZnS as a central band that does not include the tips, and ZnS at both a tip and a central band; see Table S6 for additional information. We classify these nanorods into two groups; Group 1 contains ZnS only at tips and Group 2 includes ZnS at regions within the body of the nanorods. Figure 2 also shows the percentages of each of these groups of ZnS/Cu_1.8_S nanorods at each temperature. At temperatures of 80 and 100 °C, the percentage of nanorods with ZnS domains localized at the tip regions (Group 1) was approximately 58% (*n* = 254) and 54% (*n* = 335), respectively; these values are comparable and we consider them to be effectively the same. However, at temperatures of 120 and 140 °C, which are well above the superionic transition temperature (100 °C) of copper sulfide, the percentage of nanorods with ZnS domains localized at the tip regions (Group 1) increased significantly to approximately 96% (*n* = 255) and 95% (*n* = 310), respectively. These findings indicate that the combination of the elevated thermal energy and the superionic transition in copper sulfide facilitates the migration of ZnS domains to the tip regions, resulting in the distinct domain distribution observed at 120 and 140 °C.

**Figure 1:**
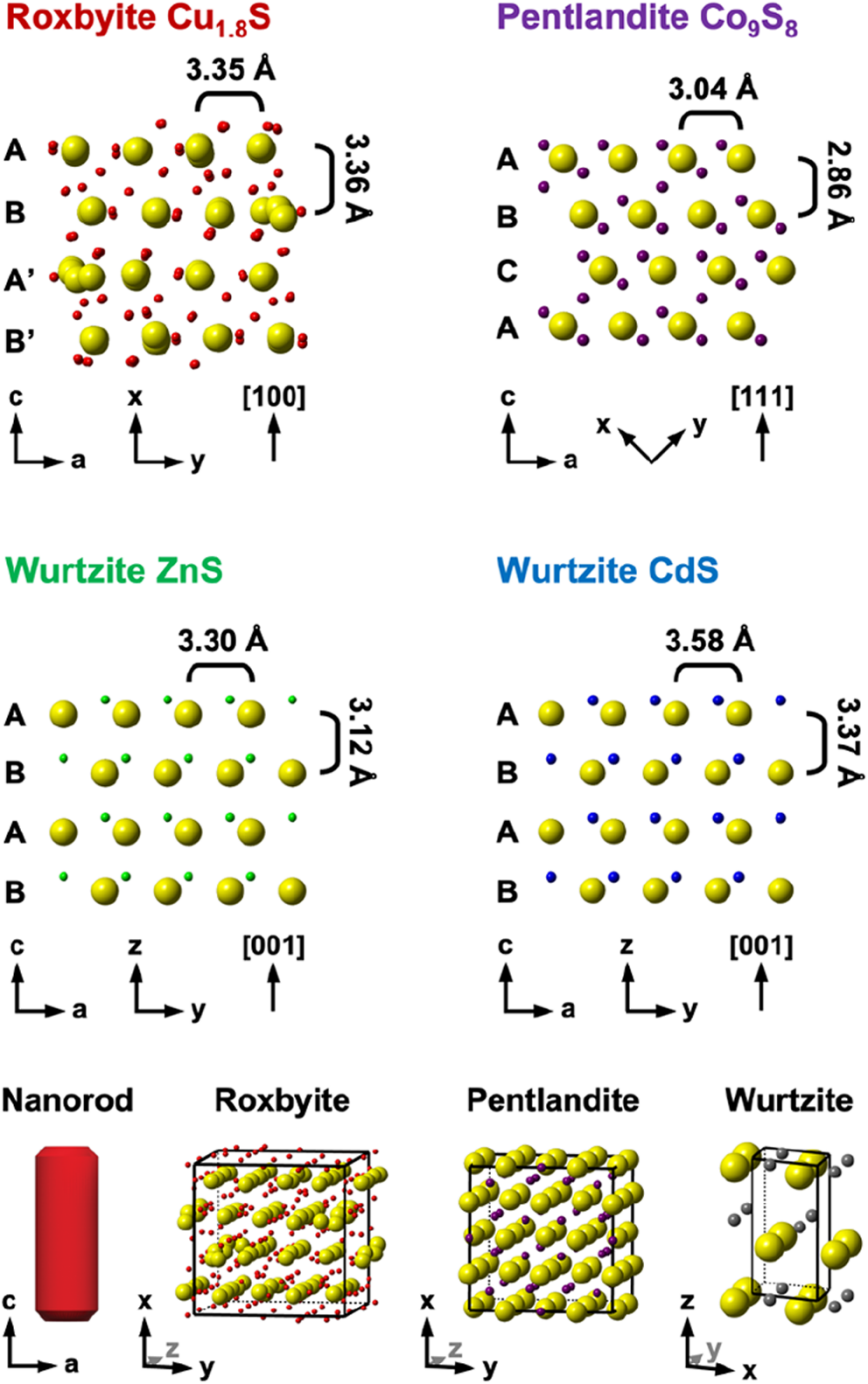
Comparison of the crystal structures of roxbyite Cu_1.8_S ([100] projection), pentlandite Co_9_S_8_ ([111] projection), wurtzite ZnS ([001] projection), and wurtzite CdS ([001] projection). The *d*-spacings that are shown were determined by average sulfur–sulfur distances along the *a*-axis and distances between sulfur–sulfur layers along the *c*-axis. The axes corresponding to the *x*, *y*, and *z* directions in the crystal structures were redefined as the *a* and *c* directions in the nanorod, which is based on the stacking of the close-packed sulfur layers of Cu_1.8_S; these designations allow for direct comparison of the oriented crystal structures in the nanorods.

**Figure 2:**
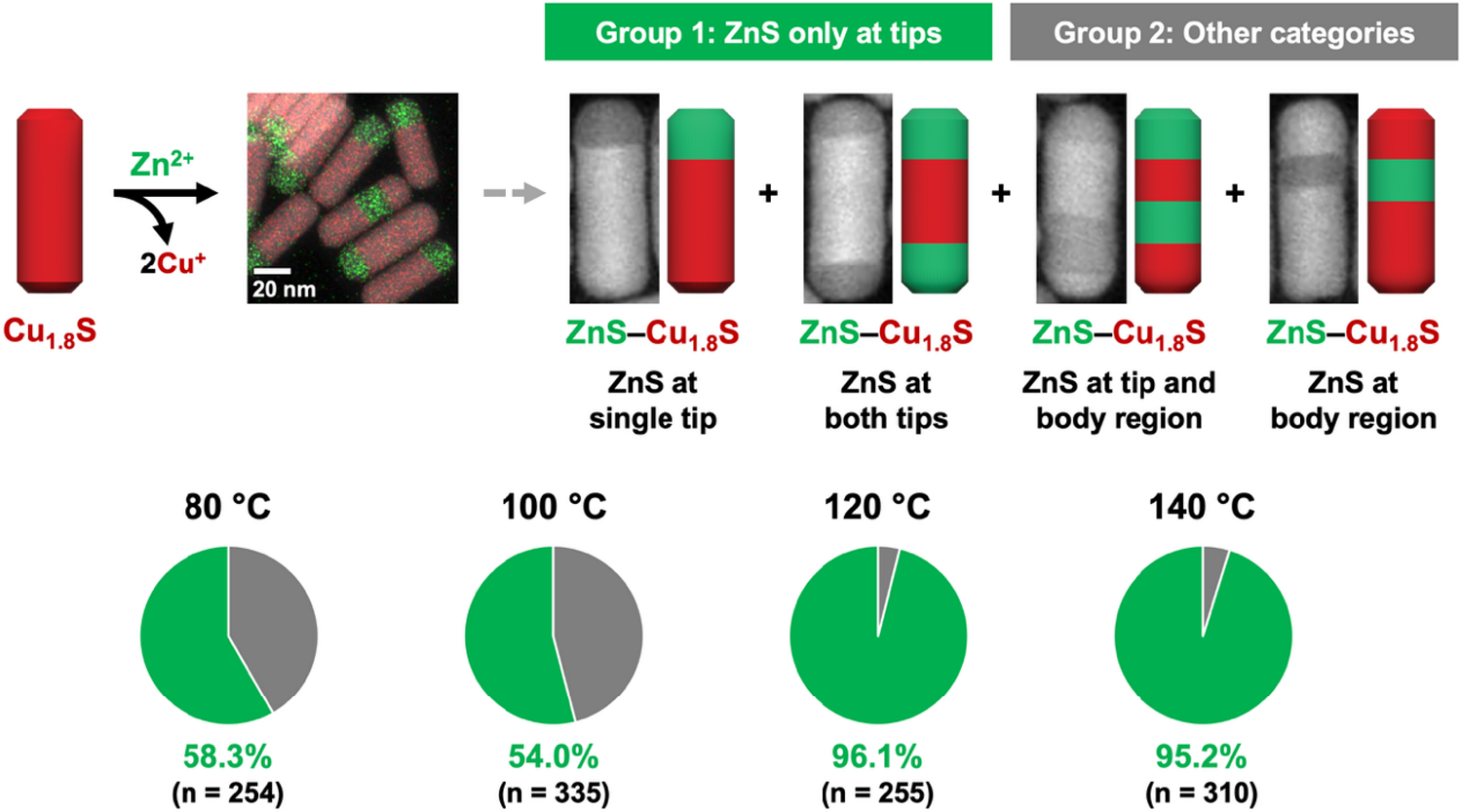
Schematic illustration and representative STEM-EDS element map for a partial Zn^2+^ exchange reaction applied to Cu_1.8_S nanorods. The resulting heterostructures were categorized into two groups based upon the spatial distributions of the ZnS domains; representative examples of each are shown as HAADF-STEM images. Group 1 (green) includes nanorods with ZnS domain(s) located exclusively at the tip(s), whereas Group 2 (gray) includes nanorods with ZnS domain(s) present in the body region. The exchange reaction was conducted over a temperature range of 80–140 °C; the resulting population distributions are summarized by pie charts, as well as statistical data. In the STEM-EDS element maps, red and green signals correspond to the Cu Kα and Zn Kα signals, respectively.

Further analysis of the subpopulation in Group 2 (Figure 2 and Table S6) reveals a subtle but meaningful insight. The nanorods containing ZnS domains at both tip and body regions were almost exclusively observed in the sample at 80 °C (8.3%, *n* = 254), whereas they were rarely found (0.2%, *n* = 900) across the samples at 100 °C, 120 °C, and 140 °C. These results suggest that at 80 °C, which is below the superionic transition temperature of copper sulfide and provides relatively low thermal energy, domain migration does not proceed efficiently during cation exchange reactions. Therefore, a small subset of nanorods develop segregated ZnS domains in both the tip and body regions. In contrast, at 100 °C, which is close to the superionic transition temperature of copper sulfide, ZnS domain migration and coalescence occurs during cation exchange, such that segregated ZnS domains at the tip and body regions are likely merged with each other to form a single domain that minimizes the number of interfaces. However, the thermal energy appears insufficient to drive the relocation of the ZnS domains to the tip.

For the Cd^2+^ exchange reactions, shown in Figure 3 and Table S2, the results at 60 and 140 °C reveal clear differences in the shapes and locations of CdS domains, despite identical reaction conditions to those used for the Zn^2+^ exchanges in Figure 2. At 60 °C, the CdS domains predominantly interface with copper sulfide along the *c*-axis, consistent with the minimization of lattice strain (Table S5).^28^ In contrast, at 140 °C, the CdS domains are frequently observed at the nanorod tips, interfacing with copper sulfide perpendicular to the *c*-axis. This alignment is energetically less favorable due to the higher lattice strain it induces compared to interfacing along the *c*-axis. These differences likely arise from the superionic behavior of copper sulfide nanorods and the increased thermal energy at 140 °C, which collectively facilitate the migration of the CdS domains toward the tips, as well as their subsequent coalescence. To further investigate the apparent tip migration behavior, we performed a control experiment in a single reaction flask, collecting aliquots at reaction temperatures of 60 and 140 °C at various reaction times (Figure S5). After Cd^2+^ exchange at 60 °C, a 30 min aging at the same temperature did not induce observable changes to the CdS domains. However, once the temperature reached 140 °C and aged for 7 min, which is the standard reaction time used for all other experiments, we already observed clear convergence of CdS domains toward the nanorod tips; extending the aging to 60 min produced no additional changes. This result confirms that *in situ* domain transformation is operable as the exchange reactions are occurring in solution.

**Figure 3:**
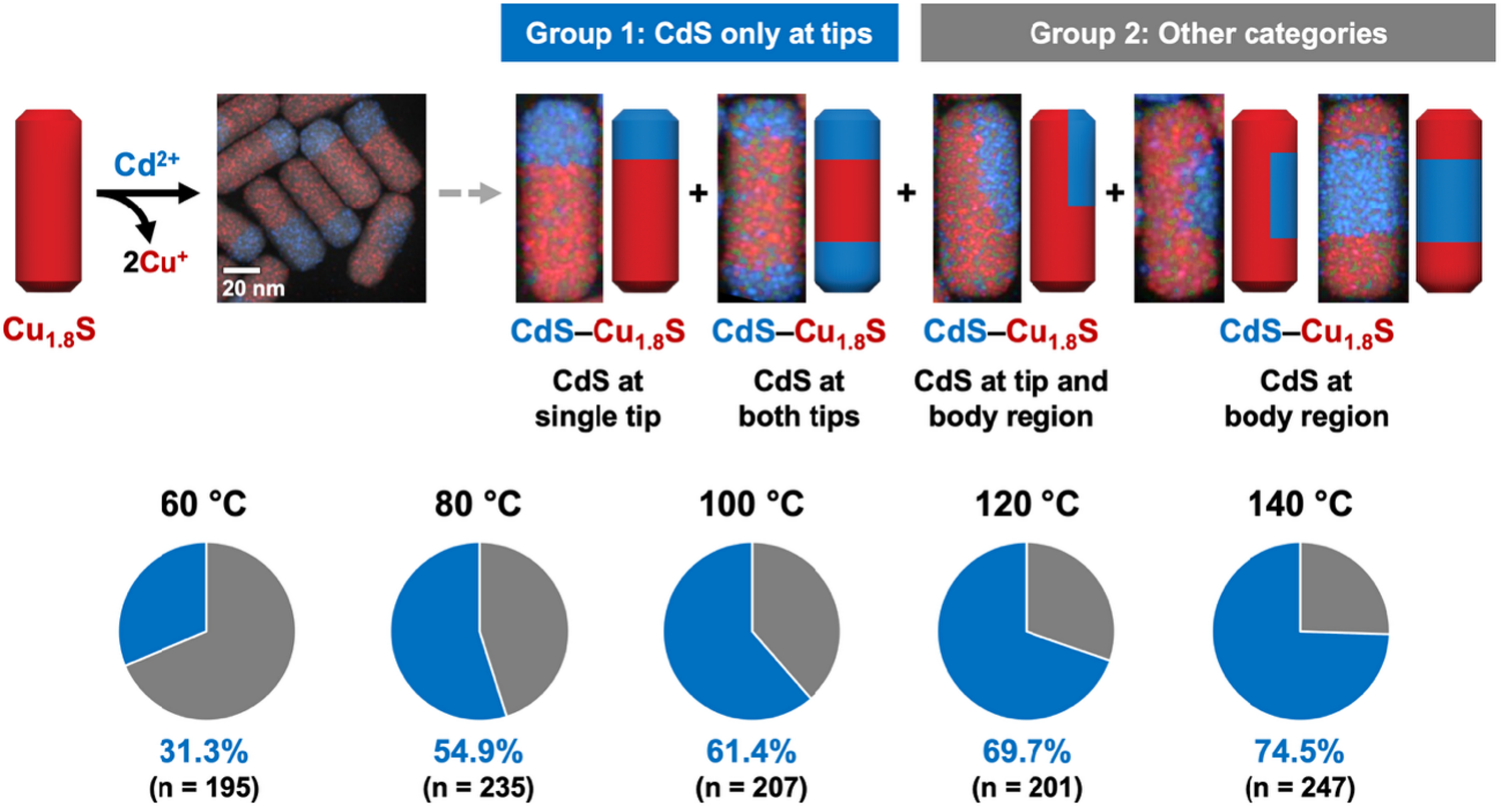
Schematic illustration and representative STEM-EDS element map for a partial Cd^2+^ exchange reaction applied to Cu_1.8_S nanorods. The resulting heterostructures were categorized into two groups based upon the spatial distributions and features of the CdS domains; representative examples of each are shown as STEM-EDS element maps. Group 1 (blue) includes nanorods with CdS domain(s) located exclusively at the tip(s), whereas Group 2 (gray) includes nanorods with CdS domain(s) present in the body region. The exchange reaction was conducted over a temperature range of 60–140 °C; the resulting population distributions are summarized by pie charts, as well as statistical data. In the STEM-EDS element maps, red and blue signals correspond to the Cu Kα and Cd Lα signals, respectively.

These distinctions of CdS domain shapes within the CdS–Cu_1.8_S heterostructured nanorods are further confirmed by XRD analysis, which reveals notable changes in relative peak intensities between the 60 °C, 100 °C, and 140 °C samples (Figure 4). In the 60 °C sample, the intensity of the (002) peak for wurtzite CdS, relative to the intensities of the (100) and (101) peaks, appears significantly higher compared to the 140 °C sample. Further inspection of the (002) peak indicates a smaller full width at half-maximum (fwhm) for the 60 °C sample. This observation indicates that the CdS domains that are aligned along the *c*-axis direction in the CdS–Cu_1.8_S nanorods,^41^ i.e., the 60 °C sample, are larger in that direction than in the 140 °C sample. Additionally, the (102) and (103) peaks have a smaller fwhm in the 60 °C sample than in the 140 °C sample; these peaks correspond to crystallographic planes that are perpendicular to the *c*-axis direction, with a slight tilt relative to the (002) plane, and further validate the larger CdS crystalline domain sizes along the *c*-axis direction in the 60 °C sample. In contrast, the (110) peak has a larger fwhm in the 60 °C sample than in the 140 °C sample. The (110) plane is tilted, but perpendicular, to the *a*-axis direction. XRD analysis for the 100 °C sample showed peak features intermediate between those of the 60 and 140 °C samples. These conclusions from XRD analysis are therefore consistent with the STEM-EDS element mapping data and confirm that the bulk of the sample matches the microscopic observations.

**Figure 4:**
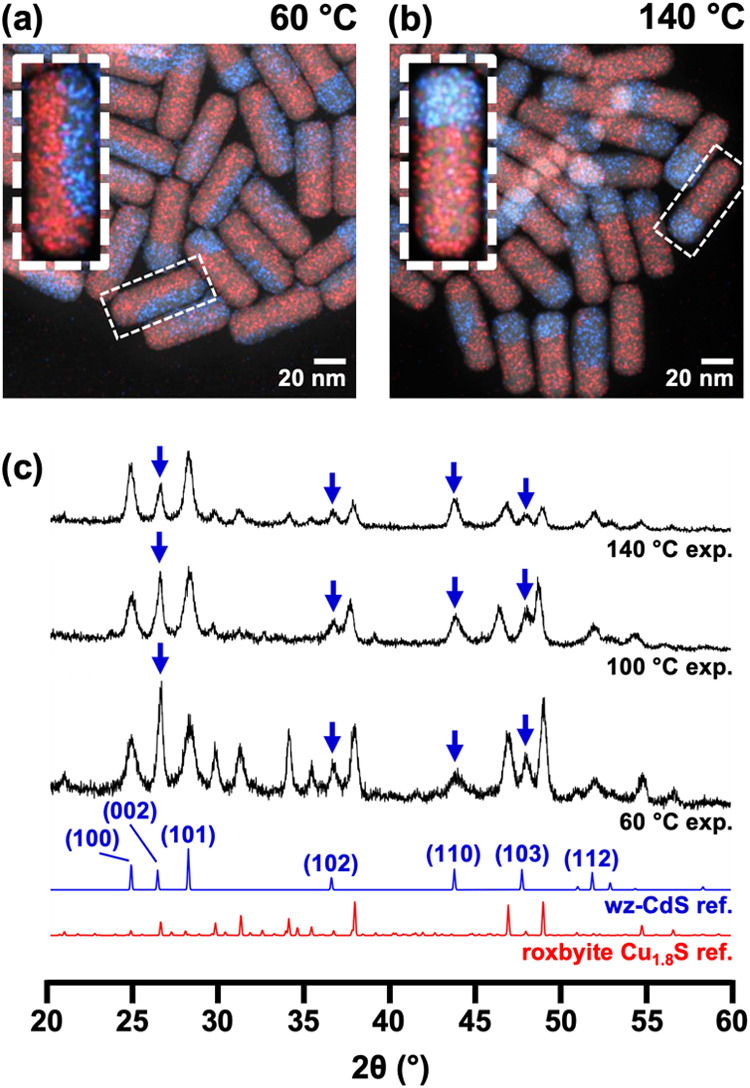
Representative STEM-EDS element maps of heterostructured nanorods acquired after partial Cd^2+^ exchange of Cu_1.8_S nanorods at reaction temperatures of (a) 60 °C and (b) 140 °C. Cropped single-nanorod STEM-EDS maps of representative heterostructure types are shown as insets. In the STEM-EDS element maps, red and blue signals correspond to the Cu Kα and Cd Lα signals, respectively. (c) XRD patterns collected from the samples shown in (a), (b), and Figure S6. Peaks marked with blue arrows indicate peaks having features that correspond to the sizes and orientations of the CdS domains, as discussed in the text. The wurtzite CdS reference pattern is from ref 42 and the roxbyite Cu_1.8_S reference pattern is from ref 43.

Returning to the CdS–Cu_1.8_S samples in Figure 3, we quantified the subpopulation of nanorods with tip-localized CdS domains at reaction temperatures of 60 °C, 80 °C, 100 °C, 120 °C, and 140 °C. The percentage of nanorods exhibiting CdS domains at the tips increased progressively with temperature (Figure 3), from 31.3% (*n* = 195) at 60 °C to 74.5% (*n* = 247) at 140 °C. Interestingly, we observed a subset of nanorods with CdS domains extending through the body of the nanorod, leaving copper sulfide regions at both tips (Figure S7). These nanorods were exclusively found in samples reacted at 120 and 140 °C (11.1%, *n* = 448), while none were observed at lower reaction temperatures of 60 °C, 80 °C, and 100 °C (0.0%, *n* = 637). At 120 and 140 °C, the combination of the superionic nature of copper sulfide and the thermal energy provide a dynamic environment that allows a subset of nanorods to overcome the lattice strain penalty (Table S3), resulting in the formation of these typically disfavored CdS/Cu_1.8_S interfaces that are near perpendicular to the *c*-axis. Population analyses throughout this study were performed using single-batch reactions. To evaluate reproducibility, we repeated a representative example experiment (Cd^2+^ exchange at 100 °C) and conducted an independent population analysis (Figure S6). The resulting fraction of nanorods exhibiting CdS domains at the tips was 52.2% (n = 209), which is comparable to the other midrange values reported in Figure 3 and therefore qualitatively consistent with the expected trend.

For Co^2+^ exchange reactions, we were unable to observe notable differences in the features of the resulting Co_9_S_8_ domains across reaction temperatures of 80 °C, 100 °C, 120 °C, and 140 °C (Table S2). In all cases, the Co_9_S_8_ domains appear within the body regions of the nanorods, including the 140 °C sample (Figure S8), consistent with the morphologies observed at lower temperatures. Additionally, at all temperatures, the Co_9_S_8_ domains interface with Cu_1.8_S along the *a*-axis direction, analogous to ZnS–Cu_1.8_S. The lattice parameter mismatch between Co_9_S_8_ and Cu_1.8_S is large along both the *a*-axis (9.7%) and *c*-axis (16%) directions (Table S5), even though the mismatch is lower along the *a*-axis, minimization of lattice mismatch is unlikely to contribute in a significant way to the placement of the Co_9_S_8_ domains within the Cu_1.8_S nanorods. Instead, comparison of the crystal structures of Co_9_S_8_ and Cu_1.8_S suggests that cation diffusion during exchange would be more favorable across the nanorods rather than along their long axes (Figure S9). Additionally, it is known that the formation of Co_9_S_8_ requires lateral shifting of the close-packed sulfur layers relative to Cu_1.8_S, transforming the distorted hcp (d-hcp) structure of Cu_1.8_S to the ccp structure of Co_9_S_8_.^18,20^ Migrating Co_9_S_8_ (ccp) throughout the Cu_1.8_S nanorods (d-hcp) would require dynamic structural shifts, based on crystal structure considerations, but this is not the case for wurtzite ZnS and CdS, which are both hcp. These crystallographic considerations indirectly help to rationalize why Co_9_S_8_ domains do not appear to migrate and coalesce above the superionic transition temperature of Cu_1.8_S, whereas ZnS and CdS do.

Building on the findings described above, we next expanded our investigation of how the superionic transition in copper sulfide and thermal energy factors influence the formation of cation-exchanged domains in heterostructured metal sulfide nanorods. Compared with Cu_1.8_S nanorods, the application of cation exchange reactions to M_*x*_S/Cu_1.8_S heterostructured nanorods proceeds differently, primarily because these heterostructured nanorods already contain M_*x*_S–Cu_1.8_S interfaces that influence where newly exchanged domains will form during subsequent exchange steps. Specifically, it is widely observed that subsequent domains tend to locate at or near the interfaces that were pre-established by earlier exchanges, and this behavior is often rationalized by increased reactivity due to the presence of structural disorder in these interfacial regions.^24^ Here, we carried out cation exchange reactions with the same three incoming cations (Zn^2+^, Cd^2+^, and Co^2+^) on the three different compositions of heterostructured nanorods (ZnS/Cu_1.8_S, CdS/Cu_1.8_S, Co_9_S_8_/Cu_1.8_S). For these experiments, we focused on two reaction temperatures, 80 and 140 °C, to probe the combined roles of superionic transitions and thermal energy on the outcomes of sequential exchange reactions applied to heterostructured nanorods (Table S3). Our choice of 80 °C was guided by the observation that this temperature lies below the superionic transition of copper sulfide and provides lower thermal energy. In contrast, 140 °C is significantly higher than the superionic transition temperature, which enhancing both cationic mobility and thermally driven migration of newly formed domains toward the rod tips.

Figure 5 shows the results for eight sets of experiments that include all combinations of subsequent Zn^2+^ and Cd^2+^ exchanges on ZnS–Cu_1.8_S, CdS–Cu_1.8_S, and Co_9_S_8_–Cu_1.8_S (except for Zn^2+^ exchange on ZnS–Cu_1.8_S and Cd^2+^ exchange on CdS–Cu_1.8_S) at temperatures of 80 or 140 °C. We find that when Zn^2+^ or Cd^2+^ cations were exchanged on CdS–Cu_1.8_S or ZnS–Cu_1.8_S heterostructured nanorods, the newly formed wurtzite ZnS or CdS domains consistently interfaced with the pre-existing CdS or ZnS domains, regardless of whether the temperature was 80 or 140 °C. Similarly, at 80 °C, Zn^2+^ or Cd^2+^ exchange on the Co_9_S_8_–Cu_1.8_S heterostructured nanorods interfaced ZnS and CdS with the pre-existing Co_9_S_8_ domains. These results are consistent with those expected based on existing knowledge. The accompanying pie charts represent the observed fraction of heterostructured nanorods that have the expected interfaces; all are >90%. (For a detailed categorization of heterostructures, see Tables S7 and S8 in the Supporting Information.)

**Figure 5:**
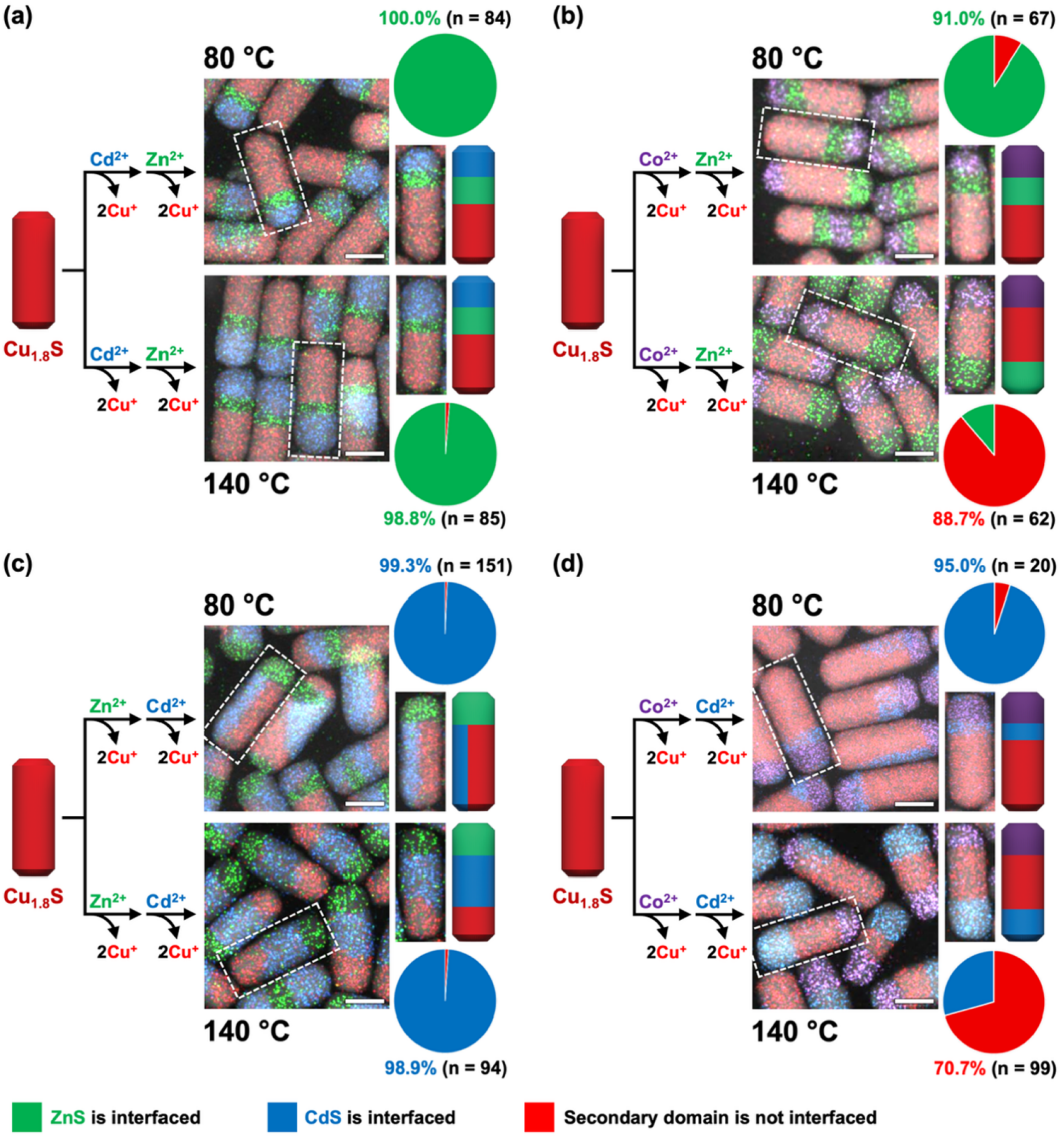
Schematic illustrations with representative HAADF-STEM images overlaid with STEM-EDS element maps for sequential exchange reactions of (a) Cd^2+^ followed by Zn^2+^, (b) Co^2+^ followed by Zn^2+^, (c) Zn^2+^ followed by Cd^2+^, and (d) Co^2+^ followed by Cd^2+^ on Cu_1.8_S nanorods at 80 and 140 °C. Representative nanorods are shown in the cropped images with corresponding schematic illustrations, and the pie charts and accompanying statistics summarize the population distributions. The green and blue portions in the pie charts indicate the percentage of nanorods in which the ZnS or CdS regions, respectively, which are the products of the second exchange, form an interface with the pre-existing domain, i.e., the product of the first exchange. The red portions indicate ZnS or CdS domains from the second exchange that are not interfaced with domains from the first exchange. In the STEM-EDS element maps, red, green, and blue signals correspond to the Cu Kα, Zn Kα, Cd Lα, and Co Kα signals, respectively. All scale bars are 20 nm.

At 140 °C, however, we observed significantly different results following subsequent Zn^2+^ or Cd^2+^ exchange of Co_9_S_8_–Cu_1.8_S heterostructured nanorods (Figure 5b,d). The newly formed ZnS or CdS domains predominantly located at the tip regions, diverging from the results obtained at lower temperatures. Such heterostructured nanorods, with Cu_1.8_S in the middle and different metal sulfides at each tip, has been observed previously in rare cases. For example, the formation of Ni_*x*_Co_9‑x_S_8_–Cu_1.8_S–ZnS nanorods, with Cu_1.8_S sandwiched between ZnS and a nickel–cobalt sulfide solid solution, was rationalized based on lattice strain considerations.^32^ However, our current results suggest that minimization of lattice strain is not the sole driving force. Instead, we propose that an elevated temperature, well above the superionic transition temperature of Cu_1.8_S, co-operates with strain effects to facilitate the migration of newly formed domains to the opposite tip. Consequently, simply tuning the reaction temperature from 80 to 140 °C transforms Co_9_S_8_–ZnS–Cu_1.8_S and Co_9_S_8_–CdS–Cu_1.8_S into Co_9_S_8_–Cu_1.8_S–ZnS and Co_9_S_8_–Cu_1.8_S–CdS, respectively. One transforms to the other as a function of increasing temperature, rather than temperature controlling the location on the nanorod from which exchange initiates and propagates. Temperature therefore provides a tuning knob for spatial control over domain placement during cation exchange reactions of metal sulfide nanoparticles.

Subsequent Co^2+^ exchanges on ZnS–Cu_1.8_S or CdS–Cu_1.8_S heterostructured nanorods similarly suggest that pre-existing interfaces influence domain placement (Figure 6a,b). At both 80 and 140 °C, newly formed Co_9_S_8_ domains appear adjacent to the ZnS or CdS regions to form ZnS–Co_9_S_8_–Cu_1.8_S and CdS–Co_9_S_8_–Cu_1.8_S, suggesting that interfacial reactivity drives material placement at low exchange temperatures. However, upon closely examining the products of Co^2+^ exchange of the ZnS–Cu_1.8_S and CdS–Cu_1.8_S heterostructured nanorods, we observed the presence of thin copper sulfide bands at the interface between ZnS and Co_9_S_8_ and at the interface between CdS and Co_9_S_8_ (Figures 6c,d, and S10–S15). This detail is important, as it indicates that ZnS/Co_9_S_8_ and CdS/Co_9_S_8_ do not actually form a direct interface, but instead form ZnS–Cu_1.8_S (or CdS–Cu_1.8_S) and Cu_1.8_S–Co_9_S_8_ interfaces. Thus, a more accurate description for these heterostructured nanorod products formed after Zn^2+^/Co^2+^ and Cd^2+^/Co^2+^ exchange are ZnS–Cu_1.8_S–Co_9_S_8_–Cu_1.8_S and CdS–Cu_1.8_S–Co_9_S_8_–Cu_1.8_S, respectively. Notably, when we carried out subsequent Zn^2+^ or Cd^2+^ exchange on Co_9_S_8_/Cu_1.8_S nanorods at 80 °C, we were unable to detect similar copper sulfide layers sandwiched between the Co_9_S_8_/ZnS and Co_9_S_8_/CdS interfaces (Figures 5b,d, S16, and S17). These observations imply that the thin copper sulfide layers form during, or as a result of, Co^2+^ exchange reactions on copper sulfide regions. Moreover, these thin copper sulfide regions appear at both 80 and 140 °C when Co^2+^ exchange is performed on ZnS–Cu_1.8_S or CdS–Cu_1.8_S heterostructured nanorods.

**Figure 6:**
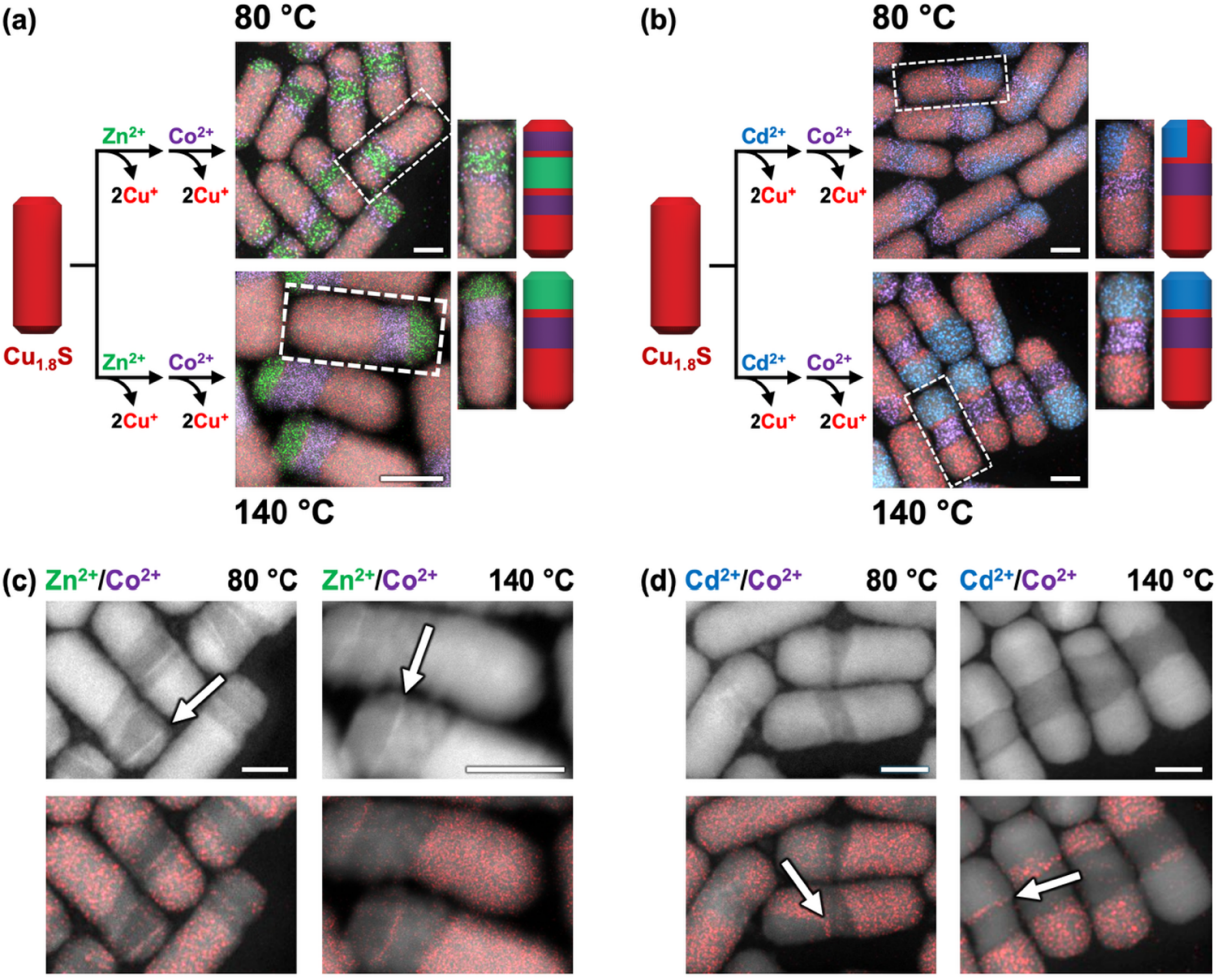
Schematic illustrations with representative HAADF-STEM images overlaid with STEM-EDS element maps for sequential exchange reactions of (a) Zn^2+^ followed by Co^2+^ and (b) Cd^2+^ followed by Co^2+^ on Cu_1.8_S nanorods at 80 and 140 °C. Representative nanorods are shown in the cropped images with corresponding schematic illustrations. In the STEM-EDS element maps, red, green, blue, and purple signals correspond to the Cu Kα, Zn Kα, Cd Lα, and Co Kα signals, respectively. HAADF-STEM images and corresponding overlaid STEM-EDS Cu Kα map (red) for exchanges involving (c) Zn^2+^ followed by Co^2+^ and (d) Cd^2+^ followed by Co^2+^, at both 80 and 140 °C. Arrows indicate the locations of the thin copper sulfide bands. All scale bars are 20 nm.

The formation of Co_9_S_8_ as the product of Co^2+^ exchange on Cu_1.8_S requires rearrangement of the sulfur sublattice structure from d-hcp to ccp, as shown in Figure 1. Shifting the entire anion sublattice laterally will be disfavored, and/or have an insufficient driving force, directly adjacent to ZnS and CdS, for which the hcp (wurtzite) and ccp (zincblende) polymorphs have comparable stability. (The zincblende polymorphs are generally more stable than wurtzite, but their energetic differences are small.) Retaining a few layers of Cu_1.8_S would help to buffer^44^ such lattice stress from the structural rearrangement required by the formation of Co_9_S_8_ upon Co^2+^ exchange. Additionally, the large lattice mismatch between Co_9_S_8_ and the other sulfides (ZnS, CdS) would disfavor a direct interface (Table S5). However, the distorted anion sublattice of Cu_1.8_S would provide localized regions of lower lattice mismatch, and therefore could relax sufficiently to preferentially form Co_9_S_8_/Cu_1.8_S interfaces rather than directly contact each other through nondistorted ccp and hcp interfaces, as would be the case for Co_9_S_8_/ZnS and Co_9_S_8_/CdS. The insights that these experiments reveal help to rationalize previously observed behavior during nanoparticle cation exchange. For example, persistent thin layers of Cu_1.8_S were observed between ZnS and Co_9_S_8_ and between CdS and Co_9_S_8_ in various types of heterostructured nanorods.^24,45^

## Conclusions

Our findings highlight how tuning reaction temperature enables control over where and how new domains form during cation exchange in heterostructured metal sulfide nanorods. By leveraging thermal energy and the superionic character of copper sulfide above ∼100 °C, which influences whether domains migrate or remain stationary,^34^ we demonstrate the formation of drastically different architectures for the same combinations of materials. This temperature-controlled versatility is especially evident in the formation of ZnS and CdS domains at the nanorod tips under higher-temperature conditions, in contrast to the interfacial growth that is observed at lower temperatures. Moreover, our results suggest that thin regions of copper sulfide emerge as buffer layers^44^ between Co_9_S_8_ and both ZnS and CdS, which help to relieve interfacial lattice strain. Collectively, these results offer mechanistic insight into how superionic transitions, lattice considerations, and partial cation exchange reactions converge to allow nuanced control over heterostructuring in multicomponent metal sulfide nanorods. These results also help to establish a foundation for designing more complex nanostructures with control over the placement of material domains and interface configurations through simple thermal control. These capabilities could be especially useful for designing semiconducting heterostructured metal sulfide nanorods capable of controlling light absorption and charge separation for applications in photocatalysis, as well as various configurations of semiconducting and metallic materials that are both electrically and optically active with tunable light emission.^1,3,8^

## Supplementary Material



## Notes

Additional experimental details and lattice constants, as well as additional XRD, HAADF-STEM, and STEM-EDS data (PDF)

## References

[1] Schaak R. E., Steimle B. C., Fenton J. L. (2020). Made-to-Order Heterostructured Nanoparticle
Libraries. Acc. Chem. Res..

[2] Drake G. A., Keating L. P., Shim M. (2023). Design Principles
of Colloidal Nanorod
Heterostructures. Chem. Rev..

[3] Bera S., Pradhan N. (2020). Perovskite Nanocrystal
Heterostructures: Synthesis,
Optical Properties, and Applications. ACS Energy
Lett..

[4] Ha M., Kim J.-H., You M., Li Q., Fan C., Nam J.-M. (2019). Multicomponent Plasmonic Nanoparticles: From Heterostructured
Nanoparticles to Colloidal Composite Nanostructures. Chem. Rev..

[5] Livakas N., Zito J., Ivanov Y. P., Otero-Martínez C., Divitini G., Infante I., Manna L. (2024). Nanocrystal Heterostructures
Based on Halide Perovskites and Metal Sulfides. J. Am. Chem. Soc..

[6] Oh N., Kim B. H., Cho S.-Y., Nam S., Rogers S. P., Jiang Y., Flanagan J. C., Zhai Y., Kim J.-H., Lee J. (2017). Double-heterojunction
nanorod light-responsive LEDs
for display applications. Science.

[7] Sitt A., Hadar I., Banin U. (2013). Band-gap engineering, optoelectronic
properties and applications of colloidal heterostructured semiconductor
nanorods. Nano Today.

[8] Zhang Y., Zhu X., Zhang Y. (2021). Exploring
Heterostructured Upconversion Nanoparticles:
From Rational Engineering to Diverse Applications. ACS Nano.

[9] Cabona A., Toso S., Griesi A., Rizzo M., Ferri M., Rusch P., Divitini G., Pérez-Prieto J., Galian R. E., Kriegel I., Manna L. (2025). Synthesis,
Growth Mechanism,
and Photocatalytic Properties of Metallic-Bi/Bi_13_S_18_Br_2_ Nano-Bell Heterostructures. ACS Mater. Letters.

[10] Hazarika A., Fedin I., Hong L., Guo J., Srivastava V., Cho W., Coropceanu I., Portner J., Diroll B. T., Philbin J. P. (2019). Colloidal
Atomic Layer Deposition with Stationary Reactant Phases
Enables Precise Synthesis of “Digital” II–VI
Nano-heterostructures with Exquisite Control of Confinement and Strain. J. Am. Chem. Soc..

[11] Jin Y., Hwang J., Han M.-K., Shon W., Rhyee J.-S., Kim S.-J. (2020). Size-Controlled
Au–Cu_2_Se Core–Shell
Nanoparticles and Their Thermoelectric Properties. ACS Appl. Mater. Interfaces.

[12] Wu K., Chen J., McBride J. R., Lian T. (2015). Efficient hot-electron
transfer by a plasmon-induced interfacial charge-transfer transition. Science.

[13] Sayevich V., Kim W. D., Robinson Z. L., Kozlov O. V., Livache C., Ahn N., Jung H., Klimov V. I. (2025). Inverted CdSe/PbSe Core/Shell Quantum
Dots with Electrically Accessible Photocarriers. ACS Energy Lett..

[14] Hou C., Wang J., Du W., Wang J., Du Y., Liu C., Zhang J., Hou H., Dang F., Zhao L., Guo Z. (2019). One-pot synthesized
molybdenum dioxide–molybdenum carbide
heterostructures coupled with 3D holey carbon nanosheets for highly
efficient and ultrastable cycling lithium-ion storage. J. Mater. Chem. A.

[15] De
Trizio L., Manna L. (2016). Forging Colloidal Nanostructures
via Cation Exchange Reactions. Chem. Rev..

[16] Li X., Ji M., Li H., Wang H., Xu M., Rong H., Wei J., Liu J., Liu J., Chen W. (2020). Cation/Anion
Exchange Reactions toward the Syntheses of Upgraded Nanostructures:
Principles and Applications. Matter.

[17] Butterfield A. G., Alameda L. T., Schaak R. E. (2021). Emergence
and Control of Stacking
Fault Formation during Nanoparticle Cation Exchange Reactions. J. Am. Chem. Soc..

[18] Butterfield A. G., McCormick C. R., Veglak J. M., Schaak R. E. (2021). Morphology-Dependent
Phase Selectivity of Cobalt Sulfide during Nanoparticle Cation Exchange
Reactions. J. Am. Chem. Soc..

[19] Fenton J. L., Steimle B. C., Schaak R. E. (2019). Structure-Selective
Synthesis of
Wurtzite and Zincblende ZnS, CdS, and CuInS2 Using Nanoparticle Cation
Exchange Reactions. Inorg. Chem..

[20] Li Z., Saruyama M., Asaka T., Tatetsu Y., Teranishi T. (2021). Determinants
of crystal structure transformation of ionic nanocrystals in cation
exchange reactions. Science.

[21] Liu Y., Liu M., Yin D., Qiao L., Fu Z., Swihart M. T. (2018). Selective
Cation Incorporation into Copper Sulfide Based Nanoheterostructures. ACS Nano.

[22] Ha D.-H., Caldwell A. H., Ward M. J., Honrao S., Mathew K., Hovden R., Koker M. K. A., Muller D. A., Hennig R. G., Robinson R. D. (2014). Solid–Solid
Phase Transformations Induced through
Cation Exchange and Strain in 2D Heterostructured Copper Sulfide Nanocrystals. Nano Lett..

[23] Fenton J. L., Steimle B. C., Schaak R. E. (2018). Tunable
intraparticle frameworks
for creating complex heterostructured nanoparticle libraries. Science.

[24] Steimle B. C., Fenton J. L., Schaak R. E. (2020). Rational
construction of a scalable
heterostructured nanorod megalibrary. Science.

[25] Gariano G., Lesnyak V., Brescia R., Bertoni G., Dang Z., Gaspari R., De Trizio L., Manna L. (2017). Role of the Crystal
Structure in Cation Exchange Reactions Involving Colloidal Cu2Se Nanocrystals. J. Am. Chem. Soc..

[26] Jo S., Kim T., Lee C. H., Lee E., Jin H., Lee S. U., Lee K., Baik H., Park J. (2025). Layer-by-Layer Interdigitated CuS/Au_2_S Heteronanoplates
by Selectively Blocking the Pathway of
Cation Exchange Reaction. J. Am. Chem. Soc..

[27] Steimle B. C., Lord R. W., Schaak R. E. (2020). Phosphine-Induced Phase Transition
in Copper Sulfide Nanoparticles Prior to Initiation of a Cation Exchange
Reaction. J. Am. Chem. Soc..

[28] Fenton J. L., Steimle B. C., Schaak R. E. (2018). Exploiting
Crystallographic Regioselectivity
To Engineer Asymmetric Three-Component Colloidal Nanoparticle Isomers
Using Partial Cation Exchange Reactions. J.
Am. Chem. Soc..

[29] Jeong C.-H., McCormick C. R., Schaak R. E. (2025). Solid Solution Formation from Sequential
Interfacial Reactions during Nanoparticle Cation Exchange. Chem. Mater..

[30] Guo M., Talebian-Kiakalaieh A., Xia B., Hu Y., Chen H., Ran J., Qiao S.-Z. (2023). Cu_7_S_4_/M_x_S_y_ (M = Cd, Ni, and
Mn) Janus Atomic Junctions for Plasmonic Energy
Upconversion Boosted Multi-Functional Photocatalysis. Adv. Funct. Mater..

[31] Park J., Park J., Lee J., Oh A., Baik H., Lee K. (2018). Janus Nanoparticle Structural Motif
Control via Asymmetric Cation
Exchange in Edge-Protected Cu_1.81_S@Ir_x_S_y_ Hexagonal Nanoplates. ACS Nano.

[32] McCormick C. R., Katzbaer R. R., Steimle B. C., Schaak R. E. (2023). Combinatorial cation
exchange for the discovery and rational synthesis of heterostructured
nanorods. Nat. Synth..

[33] Steimle B. C., Fagan A. M., Butterfield A. G., Lord R. W., McCormick C. R., Di Domizio G. A., Schaak R. E. (2020). Experimental Insights into Partial
Cation Exchange Reactions for Synthesizing Heterostructured Metal
Sulfide Nanocrystals. Chem. Mater..

[34] Young H. L., Gomez E. D., Schaak R. E. (2023). Thermally Induced Domain Migration
and Interfacial Restructuring in Cation Exchanged ZnS–Cu_1.8_S Heterostructured Nanorods. J. Am.
Chem. Soc..

[35] Lesnyak V., Brescia R., Messina G. C., Manna L. (2015). Cu Vacancies Boost
Cation Exchange Reactions in Copper Selenide Nanocrystals. J. Am. Chem. Soc..

[36] Gong J., Jain P. K. (2019). Room-temperature
superionic-phase nanocrystals synthesized
with a twinned lattice. Nat. Commun..

[37] Miller T. A., Wittenberg J. S., Wen H., Connor S., Cui Y., Lindenberg A. M. (2013). The mechanism of ultrafast structural switching in
superionic copper (I) sulphide nanocrystals. Nat. Commun..

[38] Hong Y., Venkateshalu S., Jeong S., Park J., Lee K. (2023). Regiospecific
Cation Exchange in Nanocrystals and Its Potential in Diversifying
the Nanostructural Library. Small Sci..

[39] Nelson A., Ha D.-H., Robinson R. D. (2016). Selective
Etching of Copper Sulfide
Nanoparticles and Heterostructures through Sulfur Abstraction: Phase
Transformations and Optical Properties. Chem.
Mater..

[40] Borys A. M. (2023). An Illustrated
Guide to Schlenk Line Techniques. Organometallics.

[41] Holder C. F., Schaak R. E. (2019). Tutorial on Powder X-ray Diffraction for Characterizing
Nanoscale Materials. ACS Nano.

[42] Ohata K., Saraie J., Tanaka T. (1973). Phase Diagram of the CdS-CdTe Pseudobinary
System. Jpn. J. Appl. Phys..

[43] Mumme W. G., Gable R. W., Petříček V. (2012). The Crystal
Structure of Roxbyite, Cu58S32. Can. Mineral..

[44] Nakamura S. N. S. (1991). GaN
Growth Using GaN Buffer Layer. Jpn. J. Appl.
Phys..

[45] Butterfield A. G., Steimle B. C., Schaak R. E. (2020). Retrosynthetic Design of Morphologically
Complex Metal Sulfide Nanoparticles Using Sequential Partial Cation
Exchange and Chemical Etching. ACS Mater. Letters.

