# Supplementary material for: Thermal Control of Material Placement in Heterostructured Metal Sulfide Nanorods
Source: Chem Mater. 2026 May 6;38(10):5039–48. doi: 10.1021/acs.chemmater.6c00154 (PMC13217545; doi:10.1021/acs.chemmater.6c00154)
Supplement: Supplementary file 1 [file cm6c00154_si_001.pdf]

SUPPORTING INFORMATION

Thermal Control of Material Placement in Heterostructured Metal Sulfide Nanorods

Chul-Hyun Jeong[1] and Raymond E. Schaak[1,2,3,*]

[1] *Department of Chemistry, The Pennsylvania State University, University Park, PA 16802, United States.*

[2] *Department of Chemical Engineering, The Pennsylvania State University, University Park, PA 16802, United States.*

[3] *Materials Research Institute, The Pennsylvania State University, University Park, PA 16802, Unites States.*

E-mail: res20@psu.edu

Table of Contents

Additional Experimental Details S2 – S3

Supplemental Tables (Tables S1 – S8) S4 – S11

Supplemental Figures (Figures S1 – S17) S12 – S28

Additional Experimental Details

Synthesis of roxbyite ($Cu_{1.8}S$) nanorods

Roxbyite ($Cu_{1.8}S$) nanorods were synthesized by modifying a published procedure.[1] For this synthesis, $Cu(NO_3)_2·3H_2O$ (281 mg), TOPO (2.9 g), ODE (15 mL), and OLAM (250 µL) were combined in a 50 mL three-neck round-bottom flask under the standard Schlenk line setup. The flask was placed under vacuum, heated to 80 °C for 30 min under stirring to allow degassing, and turned bright blue in color. During degassing, a 10:1 mixture of t-DDT:1-DDT (7.5 mL) was prepared in a 20 mL septum-capped vial and cycled three times under vacuum and Ar.

After degassing was completed, the flask was then cycled three times between vacuum and Ar, and subsequently maintained under an Ar flow by inserting a syringe needle connected to a bubbler through the rubber septum. The flask was then cooled to approximately 50 °C while a separate heating mantle was pre-heated by setting a Variac to maximum output for about 15 min. The cooled flask was then placed into the pre-heated heating mantle. Once the flask reached 80 °C, the t-DDT:1-DDT mixture (7.5 mL) was rapidly injected, turning the solution yellow. The flask reached 180 °C within 5 min after this injection and was then held at 180 °C for 25 min to complete the growth of $Cu_{1.8}S$ nanorods to the target size. Over the first 3 min at 180 °C, the solution gradually darkened from yellow to transparent brown before forming an opaque brown suspension. After 25 min, the flask was removed from the heating mantle and cooled to near room temperature in a water bath.

The final suspension was collected into centrifuge tubes using a 1:1 (v/v) mixture of IPA:acetone as an antisolvent, followed by centrifugation at 14,500 rpm (18,335 × *g*) for 2 min. The resulting precipitate was re-dispersed in toluene, with brief sonication, if necessary, washed again with a 1:1 mixture of IPA:acetone, and isolated by centrifugation. The product was finally re-dispersed in hexanes and stored in a 20 mL vial under ambient conditions for subsequent characterization and cation exchange reactions. The synthesized copper sulfide nanorods measured 55 ± 4 × 20 ± 1 nm (n = 307) (**Figure S1**).

Calculation of cation solution volume for partial exchange reactions

Throughout our experiments, partial exchange reactions were carried out by limiting the volume of the incoming cation solution. We first measured the mass of the $Cu_{1.8}S$ nanorods to determine the total moles of $Cu_{1.8}S$. To do this, we dispersed the nanorods (stored in hexanes) by sonication and transferred the resulting suspension into a pre-weighed empty 20 mL septum-capped vial (weighed without the cap). Using a Schlenk line system through the septum, we then evaporated the hexane until only the dried nanorods remained. Subtracting the known mass of the empty vial from the final measured weight gave us the mass of the $Cu_{1.8}S$ nanorods alone, without accounting for any surface ligands. Next, we then determined the desired fraction of exchange, of which the proportion of Cu^+ to be replaced by the incoming cation, and used the following general equation to calculate the required volume of cation exchange solution:

$$V_{cation\ solution} = \frac{mmol\ of\ Cu_{1.8}S \times \dfrac{1.8\ mmol\ of\ Cu^+}{mmol\ of\ Cu_{1.8}S}}{(ox.\ state\ of\ incoming\ cation) \times M_{cation\ solution}} \times fraction\ of\ exchange$$

In this equation, V_{cation} solution is the volume of the cation solution needed for partial exchange, the *oxidation state of the incoming cation* is consistently 2 (since only divalent metal cations were used), M_{cation} solution is the concentration of that solution, and the fraction of exchange represents the proportion of Cu^+ to be replaced. A fraction of 1 indicates complete (stoichiometric) replacement of Cu^+, whereas a value less than 1 indicates partial exchange. For instance, a fraction of 0.167 means we introduced a limited volume of metal chloride solution to

replace 1/6 of the Cu^+ in given $Cu_{1.8}S$ nanorods. Below is a sample calculation of the volume used for a 1/6 exchange on 14 mg of $Cu_{1.8}S$ nanorods. In practice, the actual volume of the cation solution was adjusted based on the precise mass of $Cu_{1.8}S$ obtained in each experiment, within 14 ± 0.5 mg range.

$$\mathrm{V_{cation\ solution}} = \frac{0.173\ \mathrm{mmol\ of\ Cu^+}}{(2) \times 0.015\ \mathrm{M_{cation\ solution}}} \times 0.167 = 0.96\ \mathrm{mL\ of\ cation\ solution}$$

Calculation of lattice parameter mismatch (%)

To compare the degree of lattice mismatch at the interfaces in the heterostructured nanorods, we calculated the lattice parameter mismatch (%) by assuming direct contact between each pair of crystal structures in **Figure 1** along either the a-axis or c-axis. In the equation below, P_1 and P_2 denote the lattice parameter values of the two crystal structures along the same axis. This equation was used to calculate the values reported in **Table S5**.

$$\frac{|P_1 - P_2|}{(P_1 + P_2)/2} \times 100\% = lattice\ parameter\ mismatch\ (\%)$$

Supplemental Tables

Table S1. Molar masses, masses of metal chloride salts, solvent volumes, solution temperatures, and target concentrations used for preparing cation solutions.

Metal salt	Molar mass	Mass of metal salt	Benzyl ether	OLAM	ODE	Temperature of solution	Target concentration
$ZnCl_2$	136.29 g/mol	51 mg	15 mL	8 mL	2 mL	180 °C	0.015 M
$CdCl_2$	183.31 g/mol	69 mg	15 mL	8 mL	2 mL	200 °C	0.015 M
$CoCl_2$	129.84 g/mol	49 mg	15 mL	8 mL	2 mL	180 °C	0.015 M

Table S2. Summary of experimental results for partial Zn^{2+}, Cd^{2+}, or Co^{2+} exchange reactions on $Cu_{1.8}S$ nanorods performed at various temperatures. In the HAADF-STEM images, regions appearing darker correspond to ZnS domains. Emission lines used for the STEM-EDS element maps were Cu Kα (red), Cd Lα (blue), and Co Kα (purple).

	Zn^{2+} exchanges	Cd^{2+} exchanges	Co^{2+} exchanges
**60 °C**	—	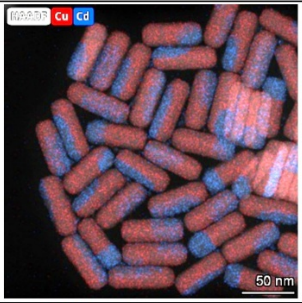	—
**80 °C**	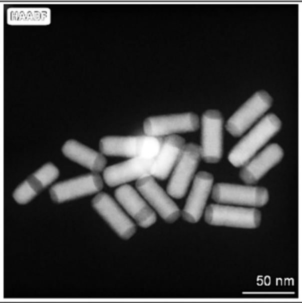	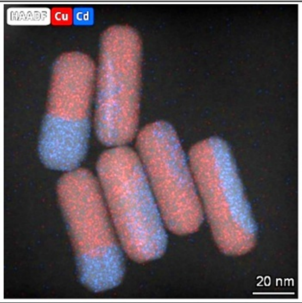	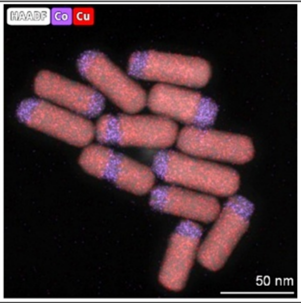
**100 °C**	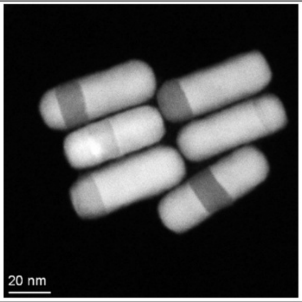	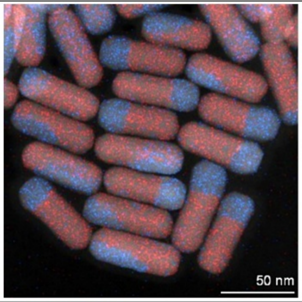	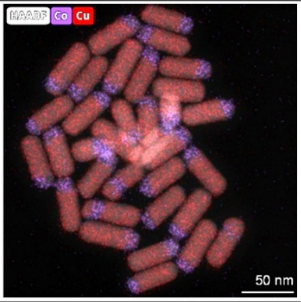
**120 °C**	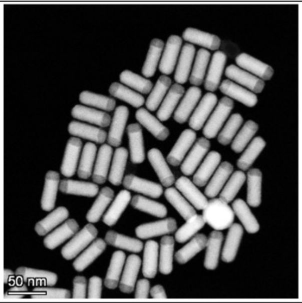	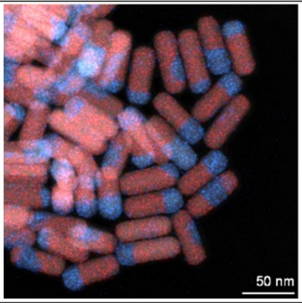	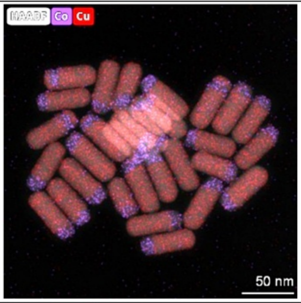
**140 °C**	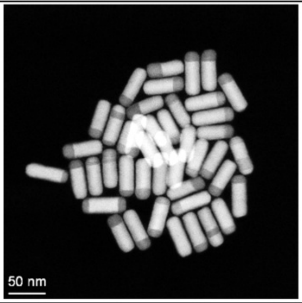	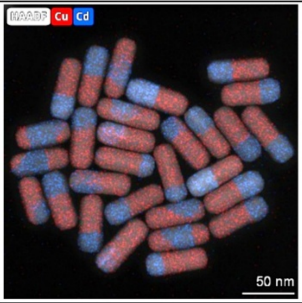	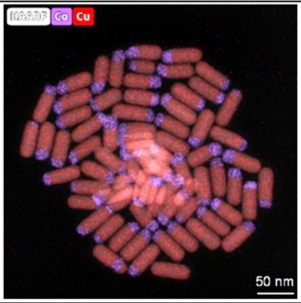

Table S3. Summary of experimental results for two-step sequential cation exchange reactions on $Cu_{1.8}S$ nanorods. Representative HAADF-STEM images and overlaid STEM-EDS element maps are shown for each reaction condition. Emission lines used in the maps were Cu Kα (red), Zn Kα (green), Cd Lα (blue), and Co Kα (purple).

<table>
<tr><th></th><th>80 °C</th><th>140 °C</th></tr>
<tr><td>1st Cd^{2+}, 2nd Zn^{2+}</td><td>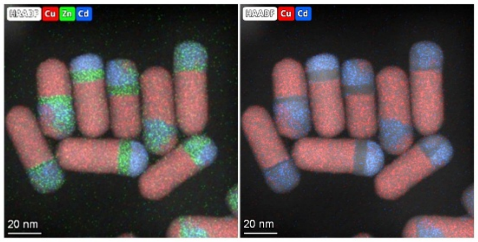</td><td>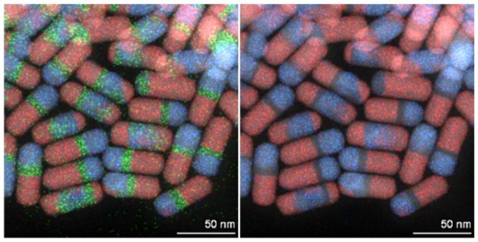</td></tr>
<tr><td>1st Co^{2+}, 2nd Zn^{2+}</td><td>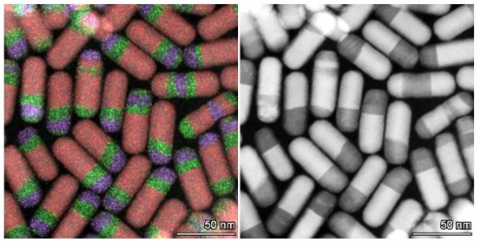</td><td>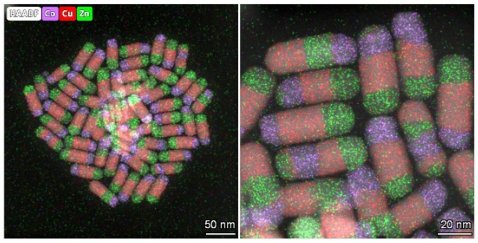</td></tr>
<tr><td>1st Zn^{2+}, 2nd Cd^{2+}</td><td>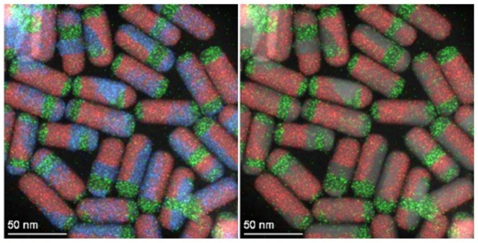</td><td>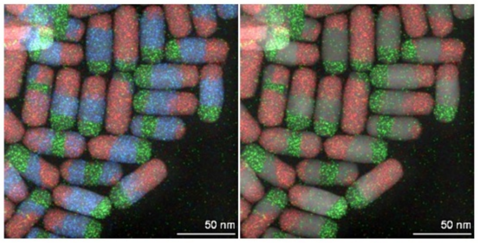</td></tr>
<tr><td>1st Co^{2+}, 2nd Cd^{2+}</td><td>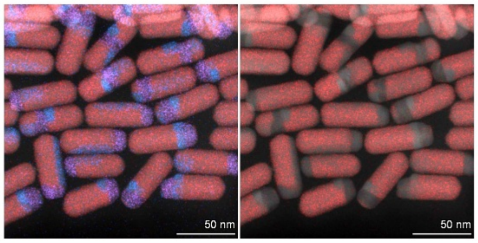</td><td>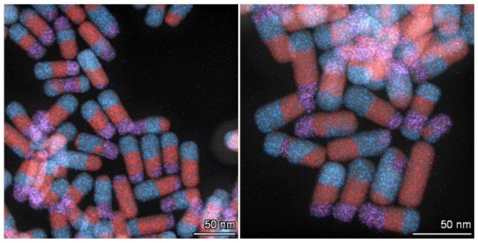</td></tr>
<tr><td>1st Zn^{2+}, 2nd Co^{2+}</td><td>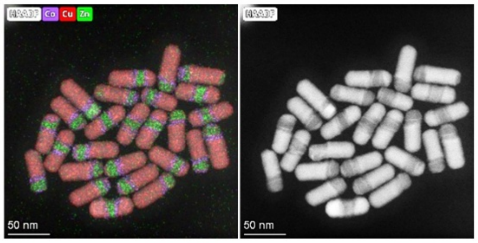</td><td>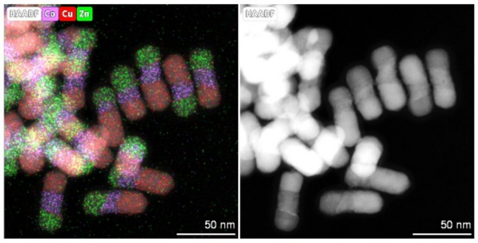</td></tr>
<tr><td>1st Cd^{2+}, 2nd Co^{2+}</td><td>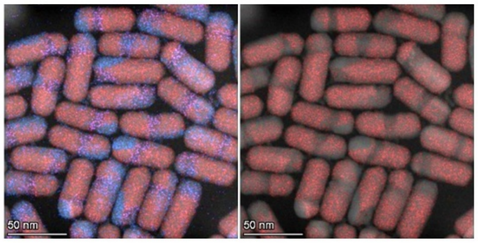</td><td>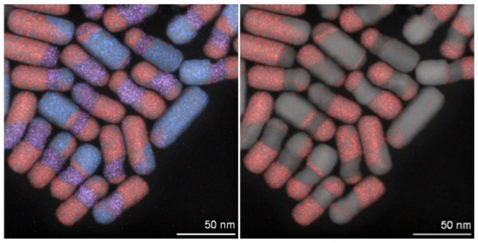</td></tr>
</table>

Table S4. Quantitative STEM-EDS analysis of partially exchanged nanorods (target 1/6 Cu^{+} replacement) obtained under different cation exchange conditions. Values represent the mean ± standard deviation obtained from three individual STEM-EDS element maps for each sample.

Partial exchanges	Zn^{2+} (80 °C)	Cd^{2+} (60 °C)	Cd^{2+} (140 °C)	Co^{2+} (80 °C)	Co^{2+} (140 °C)
Atomic fraction of M (M = Zn Kα, Cd Lα, or Co Kα)	12.7 ± 0.6%	16.1 ± 0.5%	21 ± 2%	10.9 ± 0.5%	11 ± 1%
Atomic fraction of Cu Kα	87.3 ± 0.6%	83.9 ± 0.5%	79 ± 2%	89.1 ± 0.5%	89 ± 1%

Table S5. Lattice parameter mismatch (%) between $Cu_{1.8}S$, ZnS, CdS, and Co_9S_8 calculated along the *a*-axis (*a*–*a*, perpendicular to the nanorod long axis) and the *c*-axis (*c*–*c*, parallel to the nanorod long axis), based on the values in **Figure 1**. The calculation method is described in the Additional Experimental Details section.

a – a / *c – c*	(d-hcp) $Cu_{1.8}S$	(hcp) ZnS	(hcp) CdS	(ccp) Co_9S_8
(d-hcp) $Cu_{1.8}S$		1.5%	6.6%	9.7%
(hcp) ZnS	7.4%		8.1%	8.2%
(hcp) CdS	0.3%	7.7%		16%
(ccp) Co_9S_8	16%	8.7%	16%	

Table S6. Categorization of heterostructure types obtained from partial Zn^{2+} and Cd^{2+} exchange reactions on $Cu_{1.8}S$ nanorods, shown with cropped HAADF-STEM images, overlaid STEM-EDS element maps, and schematic illustrations. Emission lines used were Cu Kα (red), and Cd Lα (blue).

	Color codes in pie charts	Descriptions	Example heterostructures with data and illustrations
Categorization of $ZnS–Cu_{1.8}S$ nanorods heterostructures	Group 1 (green): ZnS domain(s) are located at tip(s)	ZnS domain is located at one tip	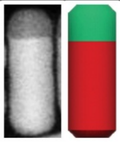
		ZnS domains are located at both tips	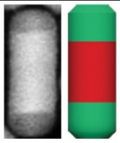
	Group 2 (gray): Other types of heterostructures	ZnS domains are located at both the tip and body regions	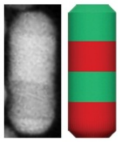
		ZnS domain is located in the body region	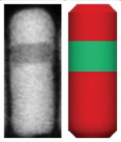
Categorization of $CdS–Cu_{1.8}S$ nanorods heterostructures	Group 1 (blue): CdS domain(s) are located at tip(s)	CdS domain is located at one tip	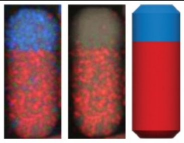
		CdS domains are located at both tips	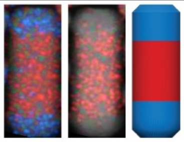
	Group 2 (gray): Other types of heterostructures	CdS domain is located in the body region, regardless of tip contact	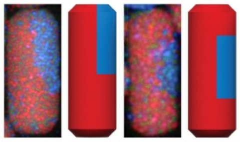
		CdS domain is located in the body region, leaving copper sulfide at the tips	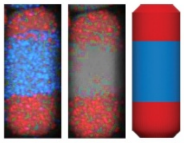

Table S7. Categorization of heterostructure types obtained from two-step sequential cation exchange reactions on $Cu_{1.8}S$ using Zn^{2+} and Cd^{2+}. Representative nanorods are shown as cropped, overlaid STEM-EDS element maps with corresponding schematic illustrations. Emission lines used were Cu Kα (red), Zn Kα (green), and Cd Lα (blue).

Reactions	Color codes in pie charts	Descriptions	Example heterostructures with data and illustrations
(80 °C) 1st Cd^{2+}, 2nd Zn^{2+}	Green: ZnS is interfaced with the pre-existing domain	ZnS is interfaced with the pre-existing CdS domain	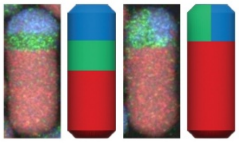
	Red: ZnS is not interfaced with the pre-existing domain	This configuration was not observed	No examples
(140 °C) 1st Cd^{2+}, 2nd Zn^{2+}	Green: ZnS is interfaced with the pre-existing domain	ZnS is interfaced with the pre-existing CdS domain	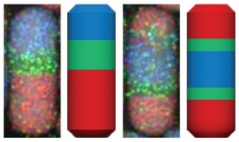
	Red: ZnS is not interfaced with the pre-existing domain	Nanorods contain ZnS in the absence of pre-existing CdS domain	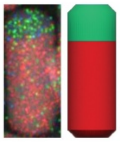
(80 °C) 1st Zn^{2+}, 2nd Cd^{2+}	Blue: CdS is interfaced with the pre-existing domain	CdS is interfaced with the pre-existing ZnS domain	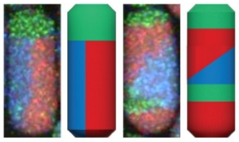
	Red: CdS is not interfaced with the pre-existing domain	Nanorods contain CdS in the absence of pre-existing ZnS domain	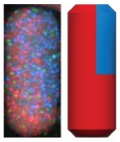
(140 °C) 1st Zn^{2+}, 2nd Cd^{2+}	Blue: CdS is interfaced with the pre-existing domain	CdS is interfaced with the pre-existing ZnS domain	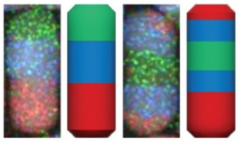
	Red: CdS is not interfaced with the pre-existing domain	Nanorods contain CdS in the absence of pre-existing ZnS domain	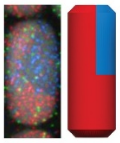

Table S8. Categorization of heterostructure types obtained from two-step sequential cation exchange reactions on $Cu_{1.8}S$ using Zn^{2+}, Cd^{2+}, and Co^{2+}. Representative nanorods are shown as cropped, overlaid STEM-EDS element maps with corresponding schematic illustrations. Emission lines used were Cu Kα (red), Zn Kα (green), Cd Lα (blue), and Co Kα (purple).

Reactions	Color codes in pie charts	Descriptions	Example heterostructures with data and illustrations
(80 °C)<br>1st Co^{2+},<br>2nd Zn^{2+}	Green: ZnS is interfaced with the pre-existing domain	ZnS is interfaced with the pre-existing Co_9S_8 domain	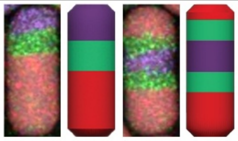
	Red: ZnS is not interfaced with the pre-existing domain	ZnS is not interfaced with the pre-existing Co_9S_8 domain	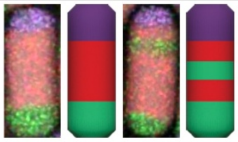
	This category is excluded from quantitative analysis	ZnS domains are present in both interfaced and non-interfaced states	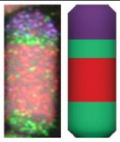
(140 °C)<br>1st Co^{2+},<br>2nd Zn^{2+}	Green: ZnS is interfaced with the pre-existing domain	ZnS is interfaced with the pre-existing Co_9S_8 domain	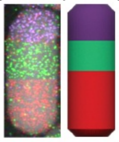
	Red: ZnS is not interfaced with the pre-existing domain	ZnS is not interfaced with the pre-existing Co_9S_8 domain	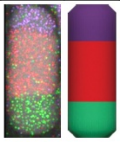
	This category is excluded from quantitative analysis	ZnS domains are present in both interfaced and non-interfaced states	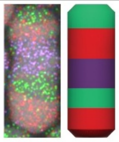
(80 °C)<br>1st Co^{2+},<br>2nd Cd^{2+}	Blue: CdS is interfaced with the pre-existing domain	CdS is interfaced with the pre-existing Co_9S_8 domain	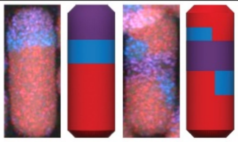
	Red: CdS is not interfaced with the pre-existing domain	CdS is not interfaced with the pre-existing Co_9S_8 domain	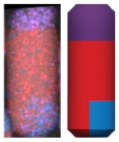
(140 °C)<br>1st Co^{2+},<br>2nd Cd^{2+}	Blue: CdS is interfaced with the pre-existing domain	CdS is interfaced with the pre-existing Co_9S_8 domain	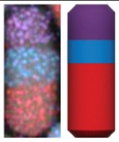
	Red: CdS is not interfaced with the pre-existing domain	CdS is not interfaced with the pre-existing Co_9S_8 domain	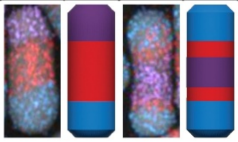

Supplemental Figures

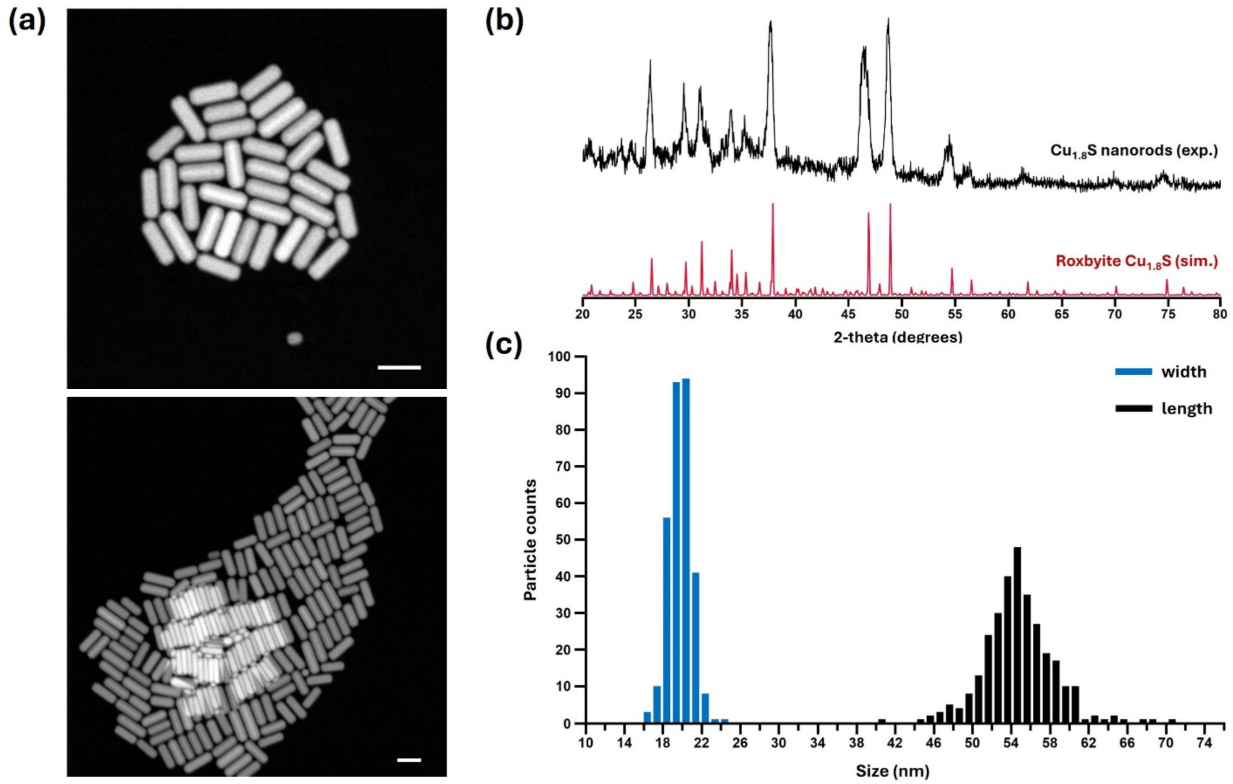

Figure S1. (a) HAADF-STEM images of $Cu_{1.8}S$ nanorods (scale bars: 50 nm). (b) Experimental powder XRD pattern of $Cu_{1.8}S$ nanorods (black) along with the reference pattern for Roxbyite $Cu_{1.8}S$ (red).[2] (c) Histogram showing the nanoparticle width (blue) and length (black). The nanorods have average sizes of 55 ± 4 nm (length) by 20 ± 1 nm (width), based on a total particle count of 307. HAADF-STEM image analysis has been carried out with ImageJ software.

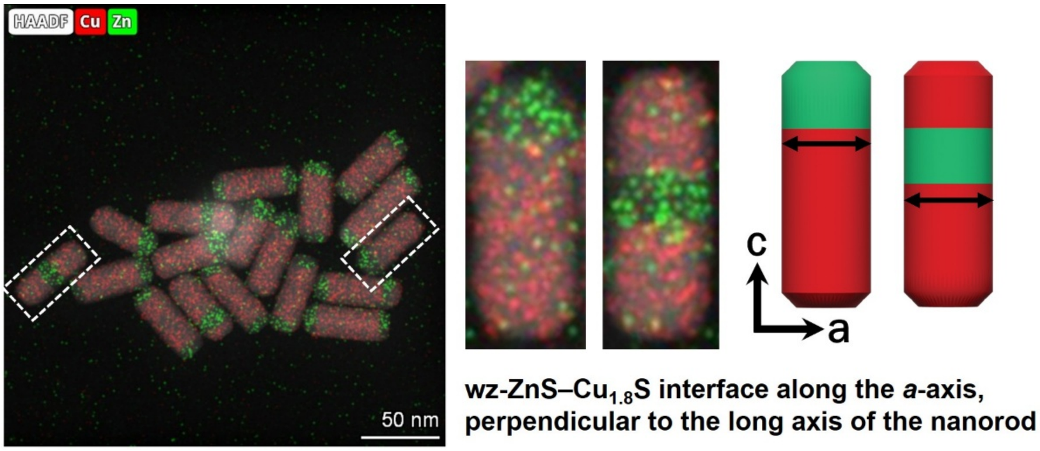

Figure S2. Overlaid STEM-EDS element maps acquired from a partial Zn^{2+} exchange reaction at 80 °C. Representative heterostructured nanorods are shown in the cropped images, along with corresponding schematic illustrations. Emission lines used were Cu Kα (red) and Zn Kα (green).

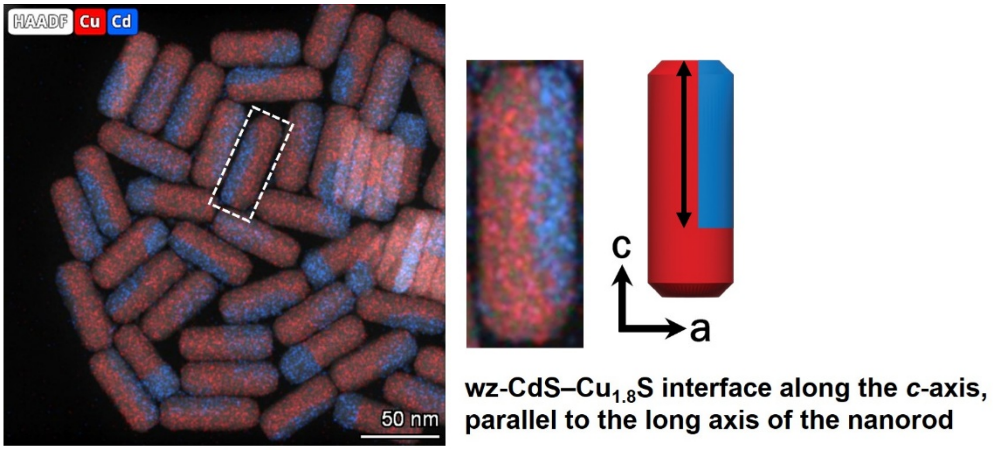

Figure S3. Overlaid STEM-EDS element maps acquired from a partial Cd^{2+} exchange reaction at 60 °C. Representative heterostructured nanorods are shown in the cropped image, along with corresponding schematic illustrations. Emission lines used were Cu Kα (red) and Cd Lα (blue).

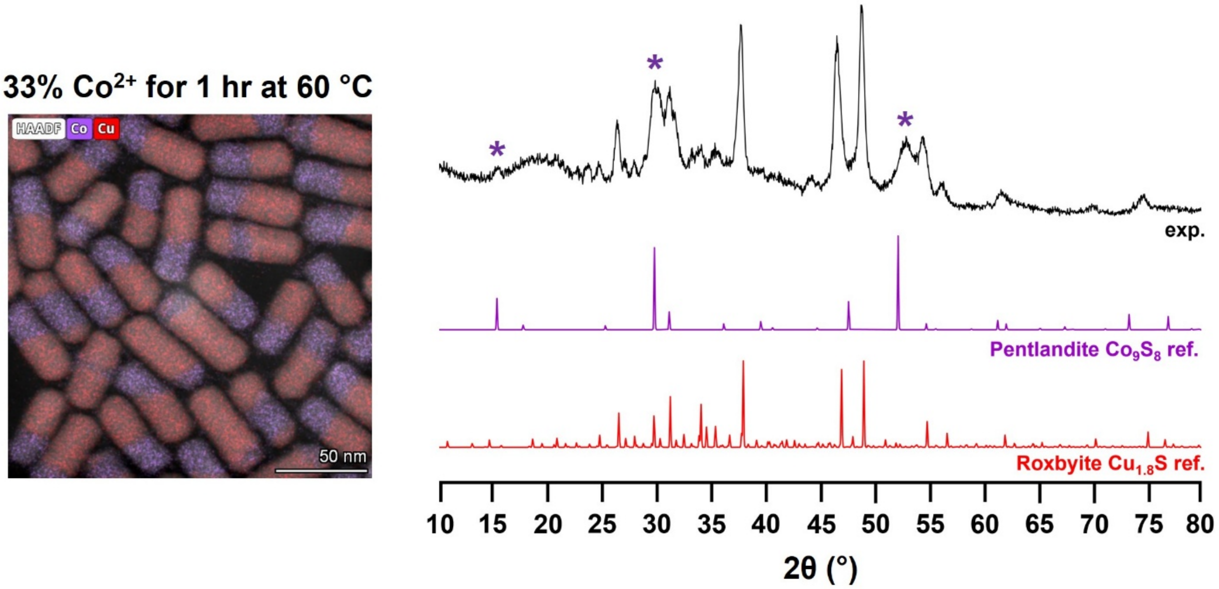

Figure S4. (Left) Overlaid STEM-EDS element maps acquired from a partial Co^{2+} exchange reaction at 60 °C. Emission lines used were Cu Kα (red) and Co Kα (purple). (Right) Experimental XRD pattern (black) of the sample shown in the STEM-EDS element map and reference patterns of pentlandite (Co_9S_8, purple)[3] and roxbyite ($Cu_{1.8}S$, red).[2]

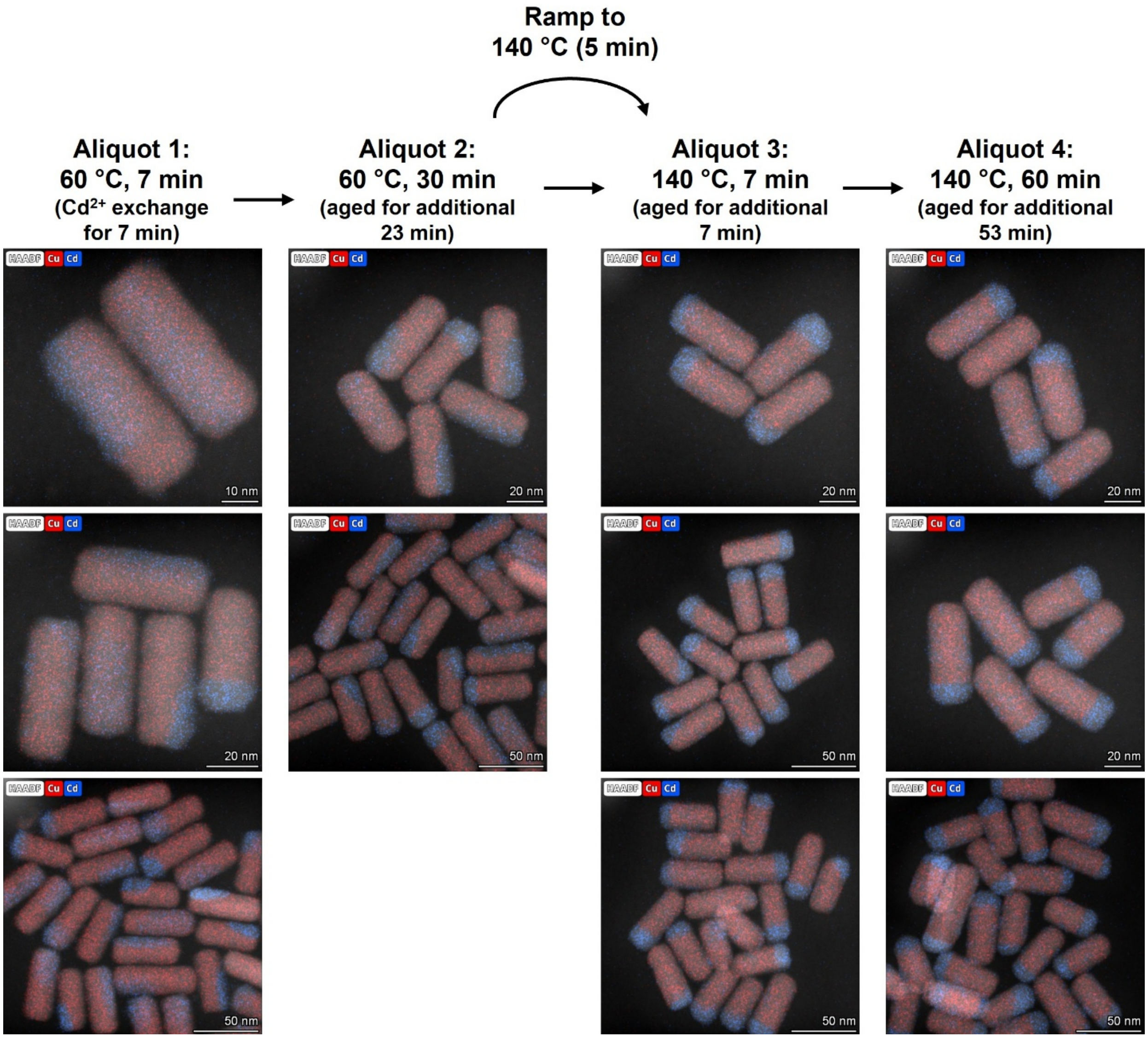

Figure S5. Overlaid STEM-EDS element maps acquired by a control experiment involving a partial Cd^{2+} exchange reaction on $Cu_{1.8}S$ nanorods. All aliquots were taken from the same single reaction flask. Emission lines used were Cu Kα (red) and Cd Lα (blue).

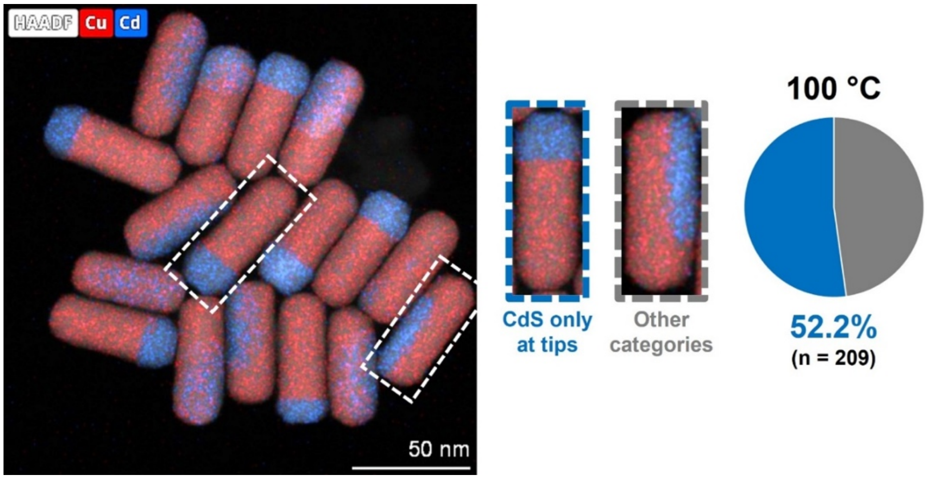

Figure S6. Overlaid STEM-EDS element map and independent population analysis from a reproduced Cd^{2+} exchange experiment at 100 °C. Emission lines used were Cu Kα (red) and Cd Lα (blue).

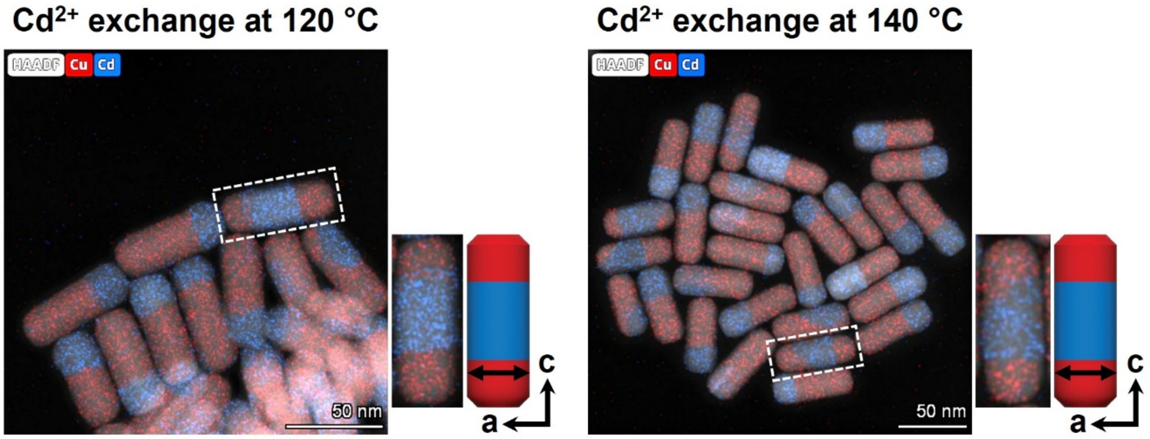

Figure S7. Overlaid STEM-EDS element maps and cropped images, along with corresponding schematic illustrations of central band-type CdS–$Cu_{1.8}S$ nanorods. Data were acquired from samples obtained by partial Cd^{2+} exchange reactions conducted at 120 °C and 140 °C. Emission lines used were Cu Kα (red) and Cd Lα (blue).

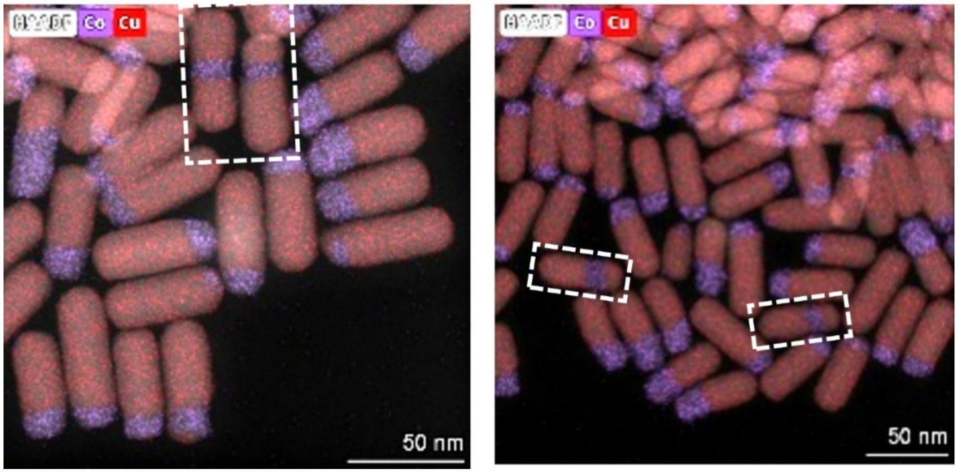

Figure S8. HAADF-STEM images with overlaid STEM-EDS element maps of heterostructured nanorods after Co^{2+} exchange at 140 °C. The dashed boxes highlight examples of nanorods exhibiting Co_9S_8 domains within the body region of the nanorods. Emission lines used were Cu Kα (red) and Co Kα (purple).

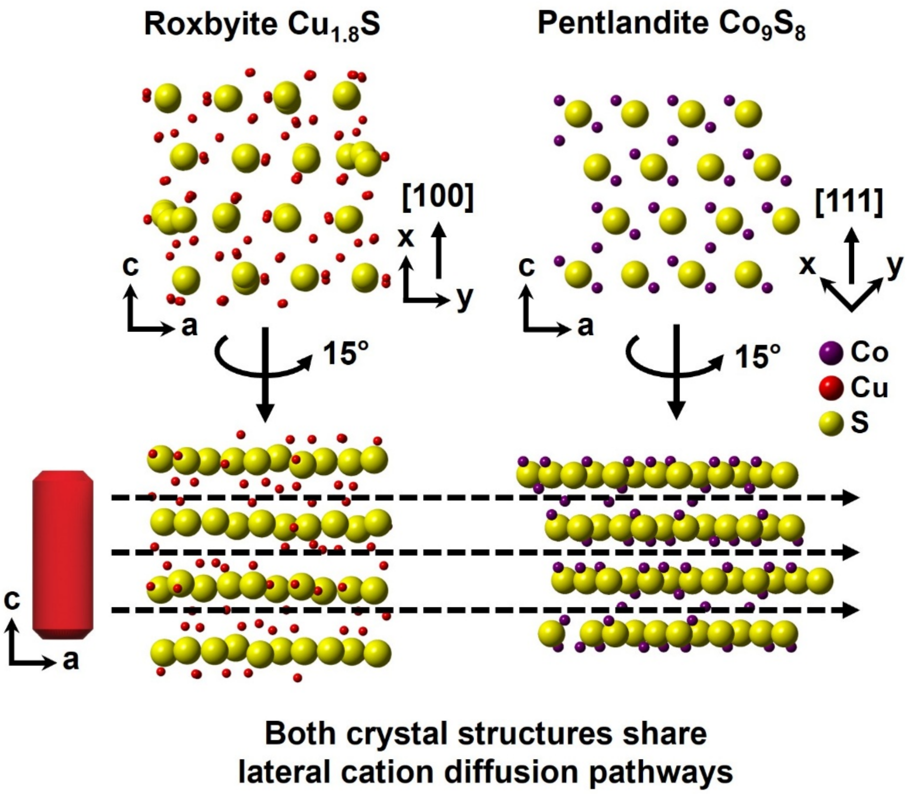

Figure S9. Structural comparison of roxbyite $Cu_{1.8}S$ and pentlandite Co_9S_8 highlighting the related lateral cation diffusion pathways perpendicular to the nanorod long axis.

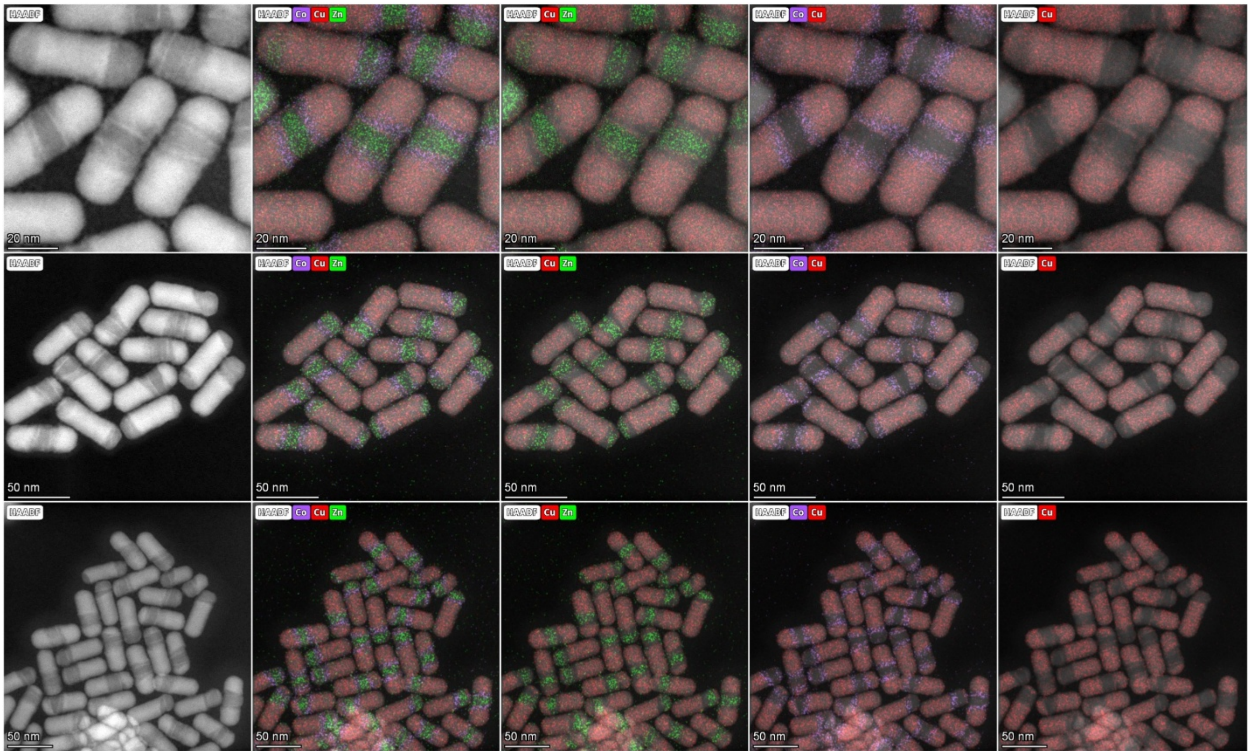

Figure S10. HAADF-STEM images and overlaid STEM-EDS element maps of heterostructured nanorods obtained from sequential cation exchange reactions (1st Zn^{2+} and 2nd Co^{2+}) conducted at 80 °C. Emission lines used were Cu Kα (red), Zn Kα (green), and Co Kα (purple).

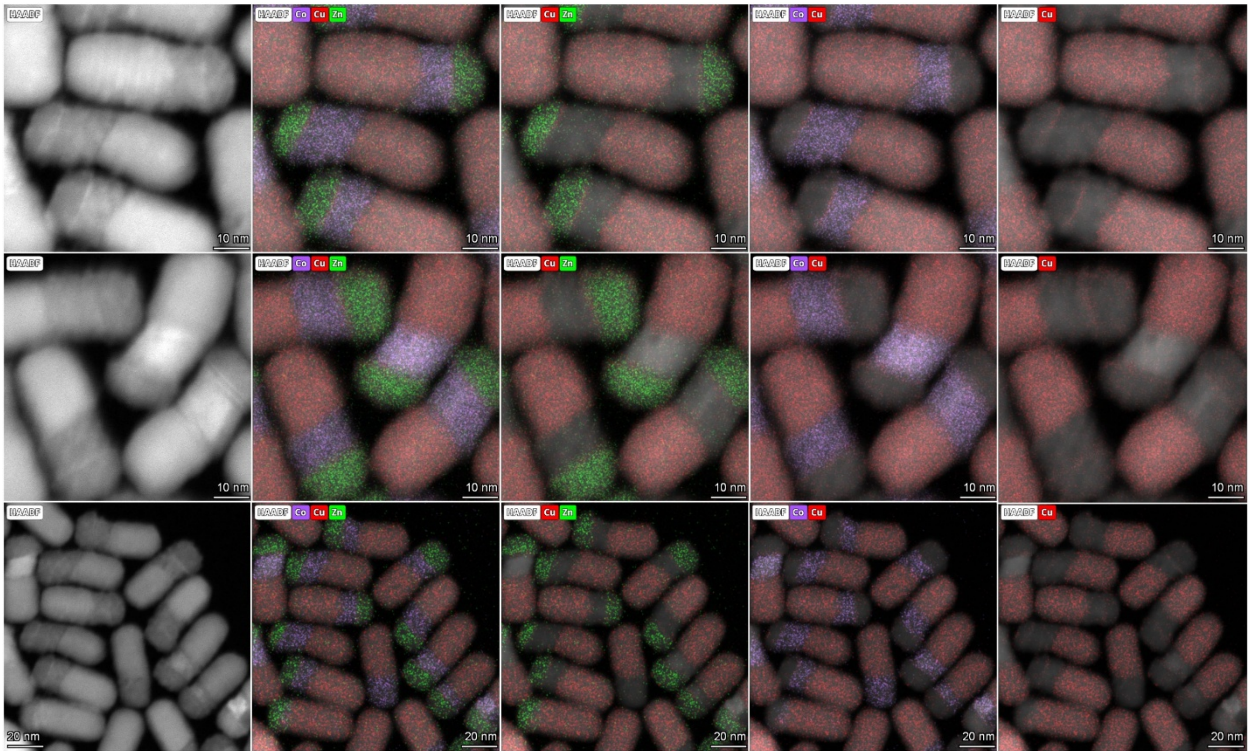

Figure S11. HAADF-STEM images and overlaid STEM-EDS element maps of heterostructured nanorods obtained from sequential cation exchange reactions (1st Zn^{2+} and 2nd Co^{2+}) conducted at 140 °C. Emission lines used were Cu Kα (red), Zn Kα (green), and Co Kα (purple).

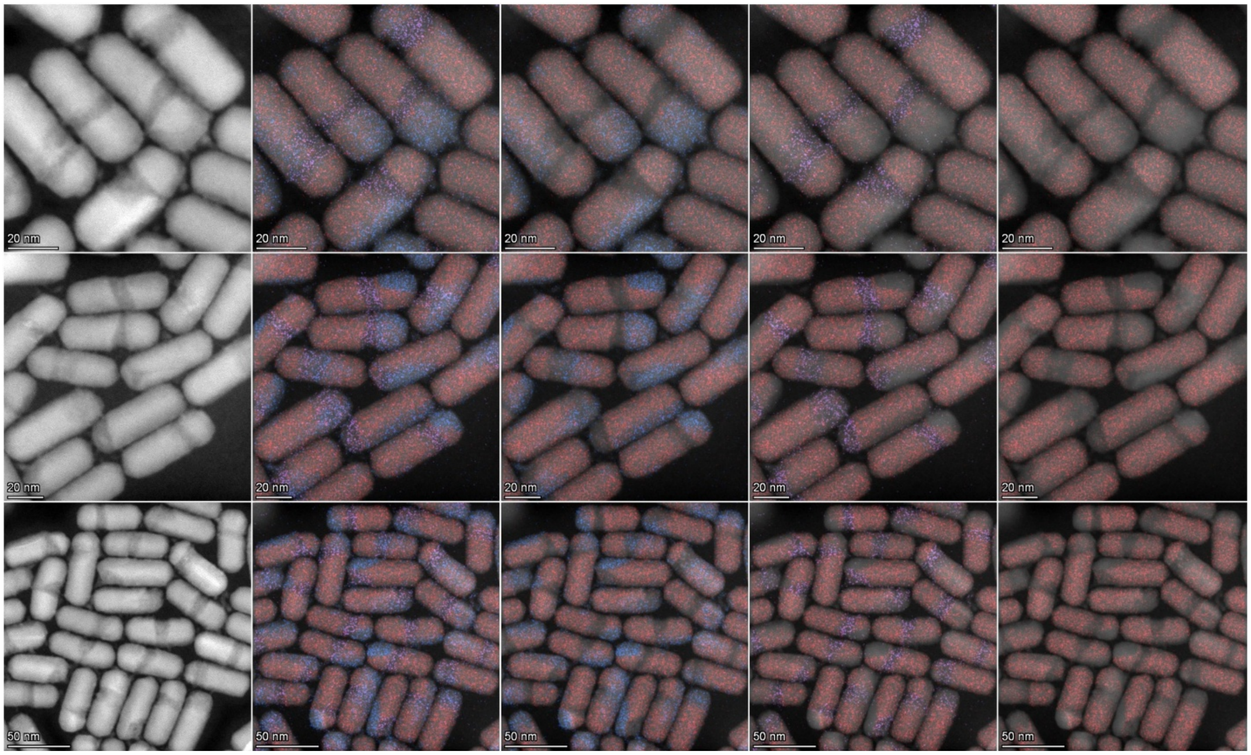

Figure S12. HAADF-STEM images and overlaid STEM-EDS element maps of heterostructured nanorods obtained from sequential cation exchange reactions (1st Cd^{2+} and 2nd Co^{2+}) conducted at 80 °C. Emission lines used were Cu Kα (red), Cd Lα (blue), and Co Kα (purple).

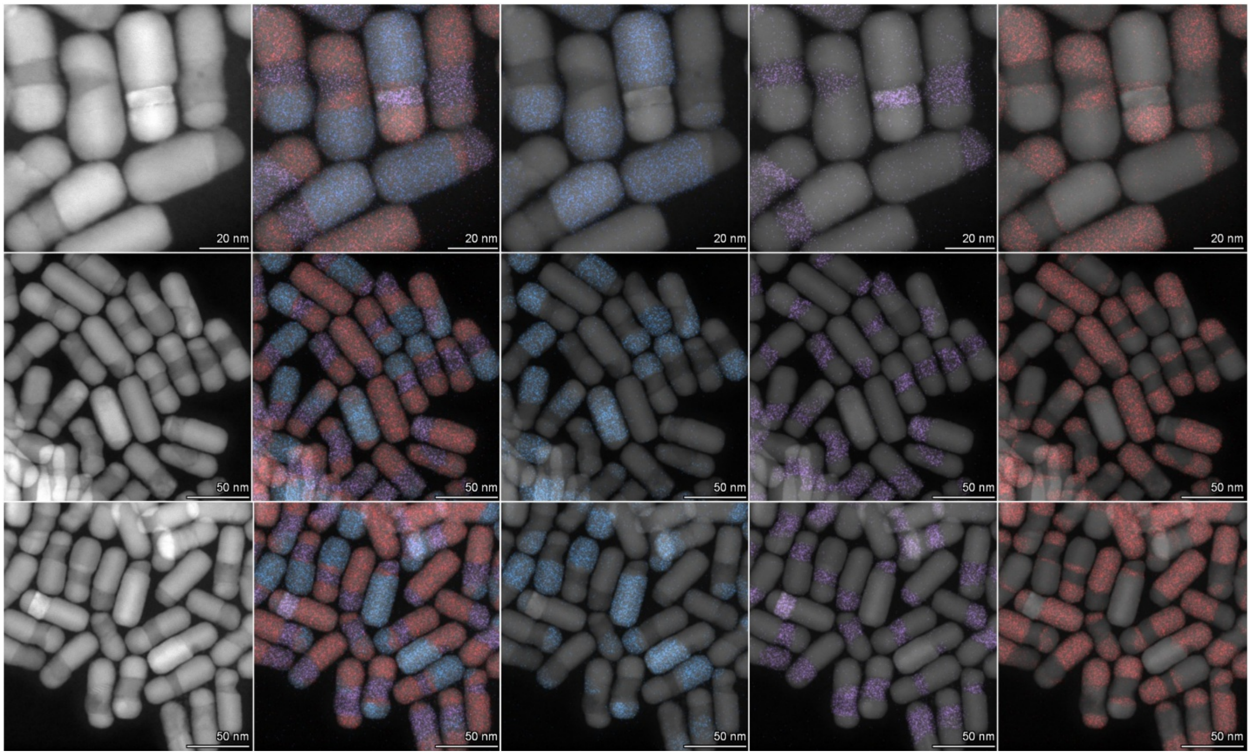

Figure S13. HAADF-STEM images and overlaid STEM-EDS element maps of heterostructured nanorods obtained from sequential cation exchange reactions (1st Cd^{2+} and 2nd Co^{2+}) conducted at 140 °C. Emission lines used were Cu Kα (red), Cd Lα (blue), and Co Kα (purple).

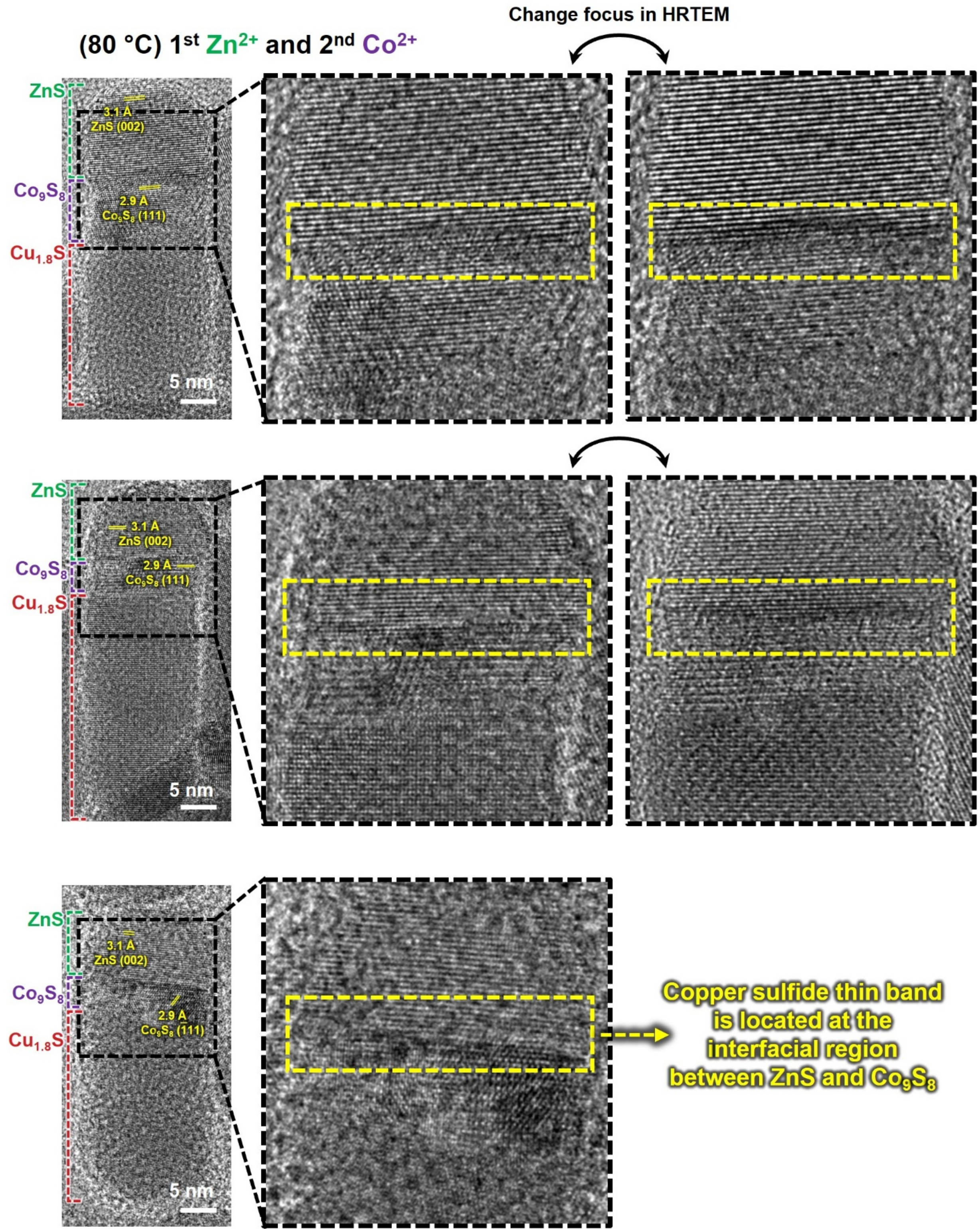

Figure S14. Magnified HRTEM images of interfacial regions in three different individual ZnS–Co_9S_8–$Cu_{1.8}S$ heterostructured nanorods obtained from a sequential exchange reaction (1st Zn^{2+} and 2nd Co^{2+}) conducted at 80°C. The focus was adjusted to observe the presence of crystalline fringes at the ZnS–Co_9S_8 interfacial regions.

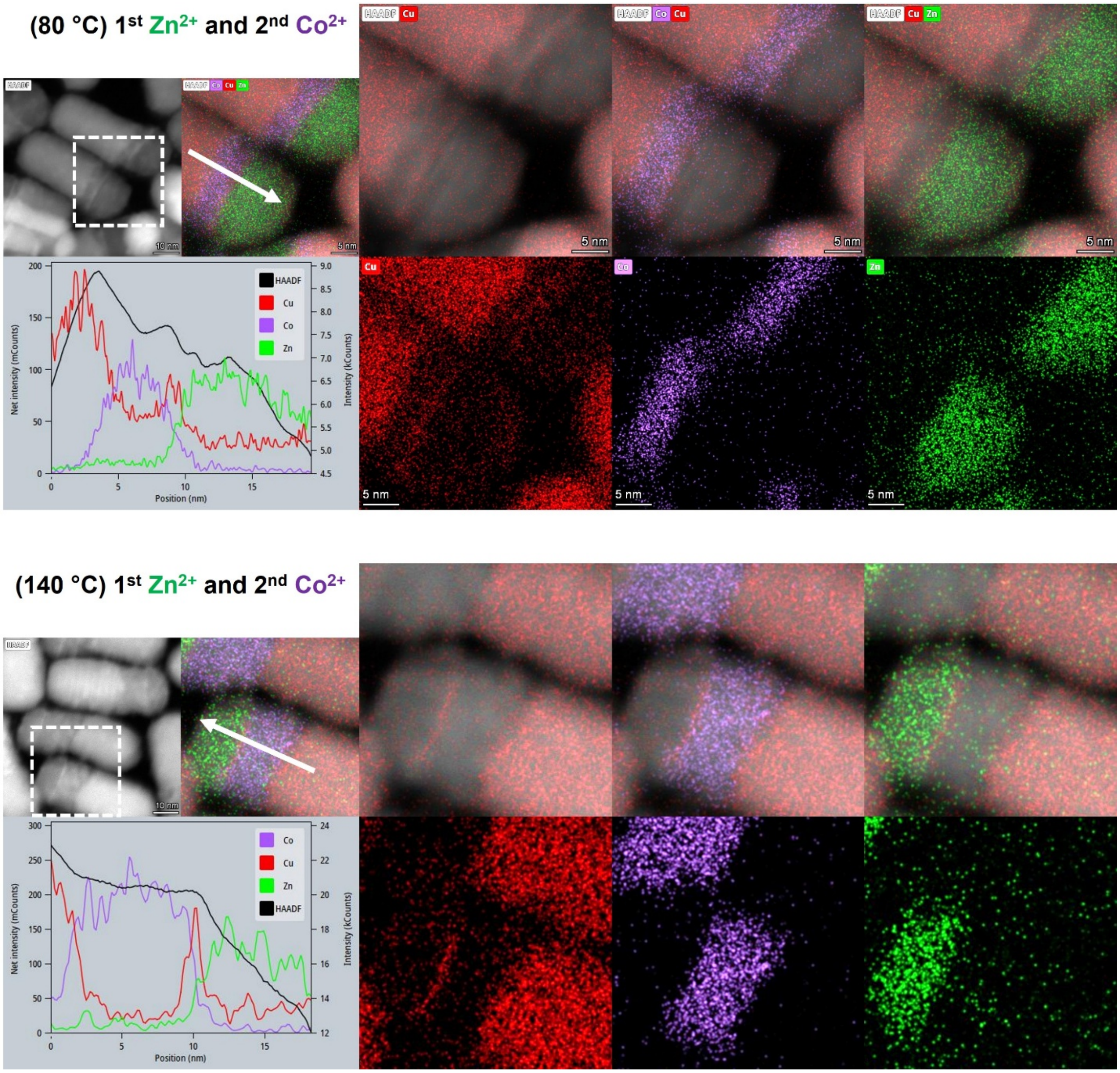

Figure S15. HAADF-STEM images and magnified overlaid STEM-EDS element maps of heterostructured nanorods after sequential exchange of Zn^{2+} followed by Co^{2+} at 80 °C (top) and 140 °C (bottom). In the STEM-EDS line-scan profiles, distinct Cu signal peaks positioned at ~9 nm (top) and ~10 nm (bottom) indicate the presence of a thin copper sulfide band between the cobalt sulfide and zinc sulfide domains. Emission lines used were Cu Kα (red), Zn Kα (green), and Co Kα (purple).

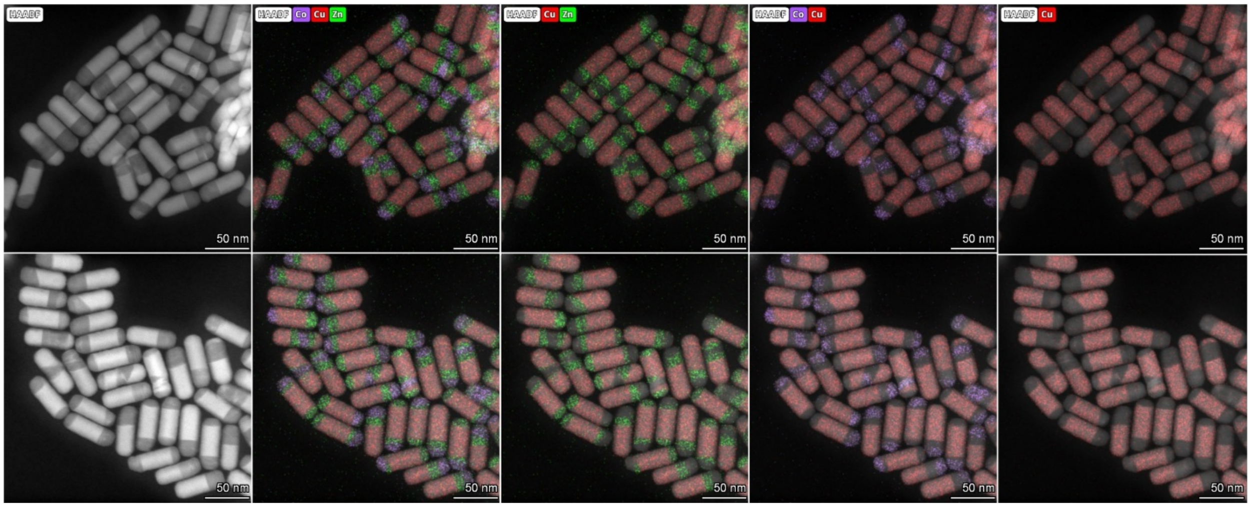

Figure S16. HAADF-STEM images and overlaid STEM-EDS element maps of heterostructured nanorods obtained from sequential cation exchange reactions (1st Co^{2+} and 2nd Zn^{2+}) conducted at 80 °C. Emission lines used were Cu Kα (red), Zn Kα (green), and Co Kα (purple).

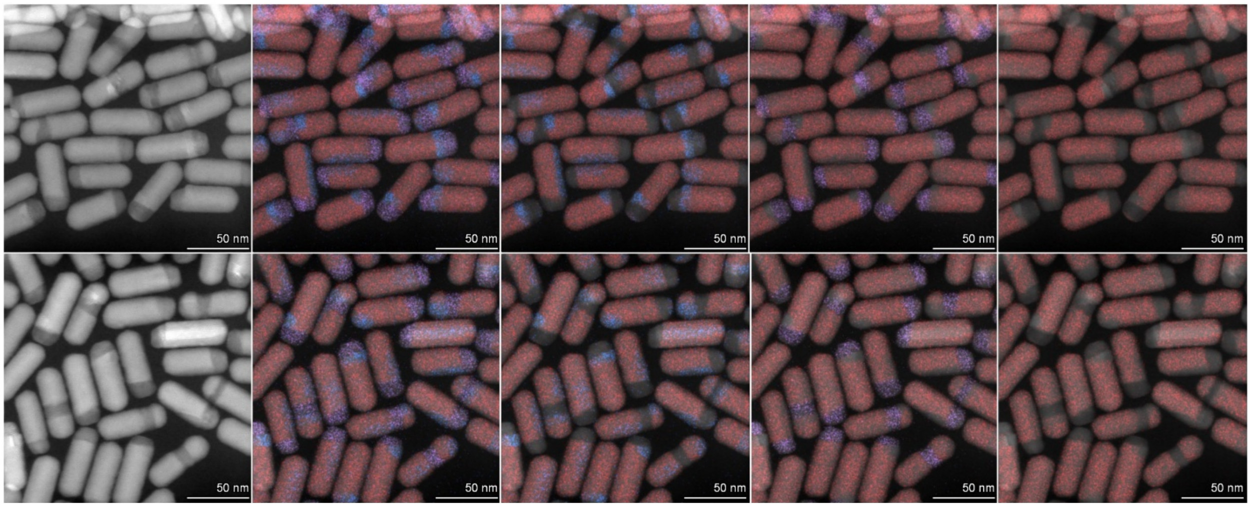

Figure S17. HAADF-STEM images and overlaid STEM-EDS element maps of heterostructured nanorods obtained from sequential cation exchange reactions (1st Co^{2+} and 2nd Cd^{2+}) conducted at 80 °C. Emission lines used were Cu Kα (red), Cd Lα (blue), and Co Kα (purple).

<u>**References**</u>

(1) Jeong, C.-H.; McCormick, C. R.; Schaak, R. E. Solid Solution Formation from Sequential Interfacial Reactions during Nanoparticle Cation Exchange. *Chem. Mater.* **2025**, *37* (20), 8291-8301. DOI: 10.1021/acs.chemmater.5c01780.

(2) Mumme, W. G.; Gable, R. W.; Petříček, V. THE CRYSTAL STRUCTURE OF ROXBYITE, $Cu_{58}S_{32}$. *Can. Mineral.* **2012**, *50* (2), 423-430. DOI: 10.3749/canmin.50.2.423.

(3) Geller, S. Refinement of the crystal structure of Co_9S_8. *Acta Crystallogr.* **1962**, *15* (12), 1195-1198. DOI: https://doi.org/10.1107/S0365110X62003187.
